# Fluorinated Trimers for Enhancing the Stability and Solubility of Organic Small Molecules Without Spectral Shifts: Ultrastable Ultraviolet Absorbers for Transparent Polyimide Films

**DOI:** 10.1002/chem.202501742

**Published:** 2025-07-02

**Authors:** Tae Gyu Hwang, Suhyeon Kim, Hong Mo Kim, Woo Jin Choi, Hyun Kyu Lee, Wan Soo Kim, Jun Ho Yoon, Yoo Sang Kim, Dong Jun Lee, Seung Yul Kim, Sang Goo Lee, Jae Pil Kim

**Affiliations:** ^1^ Interface Materials and Chemical Engineering Research Center Korea Research Institute of Chemical Technology (KRICT) Daejeon 34 114 Republic of Korea; ^2^ Semiconductor Process Architecture Team Foundry Samsung Electronics Giheung‐gu, Yongin‐si, Gyeonggi‐do 17113 Republic of Korea; ^3^ Laboratory of Organic Photo‐functional Materials Department of Materials Science and Engineering Seoul National University Seoul 08826 Republic of Korea; ^4^ Material & Component Convergence R&D Department Korea Institute of Industrial Technology (KITECH) Ansan 15588 Republic of Korea; ^5^ Division of Materials of Science and Engineering Hanyang University 222 Wangsimni‐ro, Seongdong‐gu Seoul 04763 Republic of Korea

**Keywords:** fluorinated phenyl, hydroxyphenyl benzotriazole, multimerization, solubility, stability

## Abstract

The development of highly stable organic small molecules for optical applications is of growing interest. Multimerization of monomeric small molecules can enhance stability via increased π–π stacking, but it often reduces solubility and induces undesirable spectral shifts due to π‐extension. To overcome this trade‐off, we report a trimeric small molecule (F‐TPBT) derived from a model monomer (Tinuvin 327) using a 1,3,5‐fluorinated phenyl linker. This design markedly improves thermal stability and solubility while minimizing changes in the optical absorption spectrum. F‐TPBT shows superior thermal properties (5 wt% decomposition temperature = 427 °C; weight loss after 1 hour at 300 °C = 0.32 wt%) and excellent solubility (5.3 wt% in dichloromethane; 6.4 wt% in N‐methylpyrrolidone (NMP)), with an absorption spectrum nearly identical to that of Tinuvin 327. These properties make F‐TPBT a promising UV light absorber for transparent polyimide (TPI) films, which require processing above 300 °C and are widely used in flexible displays. Notably, F‐TPBT‐containing films retain their absorption properties even after 80 hours of accelerated UV irradiation, demonstrating excellent photostability. This study presents a broadly applicable molecular design strategy for enhancing the stability and solubility of organic small molecules while avoiding significant spectral shifts.

## Graphical abstract

A molecular design strategy employing a fluorinated phenyl linker to trimerize small molecules was validated using a model small molecule (Tinuvin 327 UV absorber). The resulting trimer (F‐TPBT) showed greatly enhanced thermal stability and solubility with minimal spectral change. This strategy provides significant insight into the rational design of highly stable molecules for commercial optical applications.

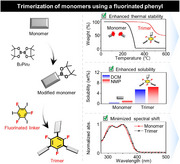

## 1 Introduction

The high stability of organic small molecules is essential for their successful integration into various commercial applications such as organic solar cells,^[^ ^1^, ^2^, ^3^ ^]^ organic light‐emitting diodes,^[^ ^4^, ^5^ ^]^ photocatalysts,^[^ ^6^, ^7^ ^]^ and flexible displays.^[^ ^8^, ^9^ ^]^ Organic small molecules possess tunable properties, including electronic behavior, solubility, thermal stability, and spectral characteristics, which can be modified through the introduction of functional groups.^[^ ^10^ ^]^ However, their intrinsic stability is considerably lower than that of inorganic materials and must be improved to enable industrial applications.^[^ ^11^ ^]^

Several strategies have been explored to enhance the stability of organic small molecules, including organic–inorganic hybridization, chemical bonding facilitation, and the enhancement of intermolecular interactions through poly‐ or multimerization.^[^ ^12^, ^13^, ^14^, ^15^, ^16^ ^]^ Among these approaches, multimerization, in which monomers are interconnected via linker units to form dimers, trimers, or tetramers, has gained increasing attention in optical applications.^[^ ^17^, ^18^, ^19^, ^20^ ^]^ However, a trade‐off between improved stability and other key properties (e.g., solubility, optical spectra, and electronic characteristics) complicates simultaneous optimization. Generally, multimerization strengthens intermolecular interactions such as π–π stacking, leading to enhanced molecular stability. However, it also increases molecular planarity, which reduces solubility, and extends electronic conjugation, which causes red‐shifted optical spectra and can alter electronic behavior.^[^ ^21^, ^22^ ^]^ Although this strategy holds great promise for the commercialization of organic small molecules in optical applications, it has so far received limited research attention and remains at an early stage of development.

Therefore, in this study, we present a promising trimerization strategy using a model organic small molecule, hydroxyphenyl benzotriazole (HBT), to simultaneously enhance its solubility and thermal stability without significant spectral changes. HBT is a functional molecule that reduces polymer photodegradation induced by ultraviolet (UV)‐A radiation (320–400 nm), but is thermally labile and therefore unsuitable for applications requiring high‐temperature processes, such as those used to produce TPI films for flexible displays.^[^ ^23^ ^]^ Herein, we show that the trimerization of a model HBT‐based UV absorber (Tinuvin 327) using a 1,3,5‐fluorinated linker enhances its stability and solubility while inducing almost no changes in its optical absorption spectrum and demonstrate the potential of the trimer as a UV absorber for high‐temperature‐processed TPI films. Moreover, UV‐visible spectroscopy, thermogravimetric analysis (TGA) coupled with gas chromatography‐mass spectrometry (TGA‐GC/MS), and density functional theory (DFT)‐based computational studies are used to investigate the effects of trimerization on optical properties, thermal stability, photostability, and solubility. Thus, this study presents a universal molecular design strategy for enhancing the reliability of organic small molecules and thus facilitating their broader adoption in industrial fields.

## 2 Experimental

All chemicals were prepared from commercial vendors and used as received. Powder X‐ray diffraction (XRD) patterns were recorded on a D8‐Advance instrument (Bruker, USA). TGA was performed using a TA instrument Q500. Ultraviolet‐visible (UV/vis) absorption spectra were recorded using a SHIMADZU UV‐1900 UV‐vis spectrophotometer. The Supporting Information provides further experimental details, including those pertaining to compound synthesis and characterization (Schemes S1–S3, Figure S1), fabrication of TPI thin films and their side‐view scanning electron microscopy images (Figure S2), ^1^H and ^13^C nuclear magnetic resonance spectra of the modified HBT monomer (M‐Pin), trimers (TPBT and F‐TPBT), and TPI (PI‐HD) (Figures S3–S10), matrix‐assisted laser desorption/ionization time‐of‐flight (MALDI‐TOF) mass spectra (Figures S11–S13), Fourier‐transform infrared (FT‐IR) spectrometer results (Figures S14, S15), Differential scanning calorimetry (DSC) results (Figures S16, S17) of different compounds, gel permeation chromatography (GPC) trace of PI‐HD (Figure S18, Table S1), and DFT calculation results obtained for Tinuvin 327, TPBT, and F‐TPBT.

## 3 Results and Discussion

**Figure 1:**
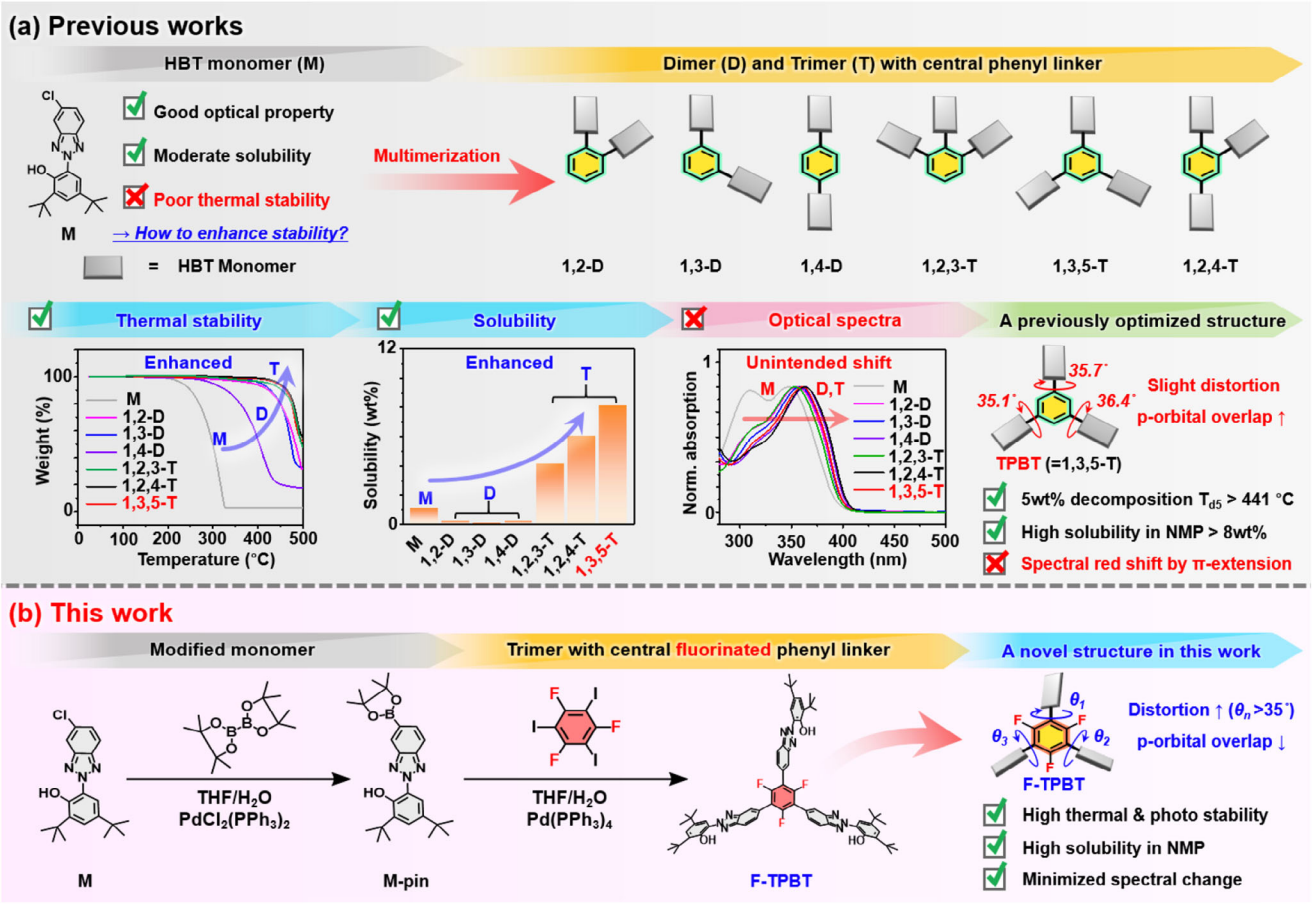
Summary of a) previous studies and b) this study.

Our previous studies (Figure 1a) focused on developing highly stable UV absorbers compatible with TPI films, which are used in flexible displays and require processing at >300 °C.^[^ ^8^, ^21^, ^23^ ^]^ The examined HBT monomer (M, see Figure 1) was shown to effectively absorb UV‐A radiation and exhibit a moderate solubility but was insufficiently thermally stable for use in TPI films. To address this issue, we enhanced the stability of M by modifying it into dimers or trimers using a phenyl linker. Three dimer (1,2‐D, 1,3‐D, and 1,4‐D) and three trimer (1,2,3‐T, 1,3,5‐T, and 1,2,4‐T) structures were synthesized and analyzed. Thermal stability depended on the number and position of M units attached to the phenyl linker, generally following the order of T > D > M. Solubility in NMP followed the order of T > M > D. Thus, the enhanced thermal stability was ascribed to the enhancement of intermolecular interactions with the increasing molecular weight. The dimers (which have a higher structural planarity than M) exhibited a markedly low solubility (<0.1 wt% in NMP), whereas the structurally distorted trimers showed a higher solubility (4–8 wt% in NMP). The 1,3,5‐T (TPBT) structure exhibited optimal properties, featuring a 5 wt% decomposition temperature of 441 °C and an NMP solubility of >8.1 wt%. All D and T structures, including TPBT, exhibited an increased p‐orbital overlap between substituted monomer units, which led to red‐shifted absorption spectra and spectral shape changes. Consequently, the use of these compounds as UV absorbers for TPI films resulted in an increased absorption at the lower end of the visible spectrum (at ∼400 nm) and reduced film transmittance.

The present study aimed to develop a highly stable and soluble UV absorber while minimizing p‐orbital overlap to reduce the spectral red shift (Figure 1b). To achieve this goal, we designed and synthesized a trimer featuring three M units attached at the 1,3,5‐positions of a fluorinated phenyl linker (F‐TPBT) via Pd‐catalyzed cross‐coupling. The linker was expected to induce considerable distortions between the substituted M units due to the steric hindrance of fluorine, thereby hindering p‐orbital overlap between these units and minimizing the spectral red shift. As a result, F‐TPBT, which is structurally similar to TPBT, was also expected to have superior stability and solubility, as the nonfluorinated counterpart of the former was reported to be exceptionally stable and soluble.^[^ ^21^ ^]^

**Figure 2:**
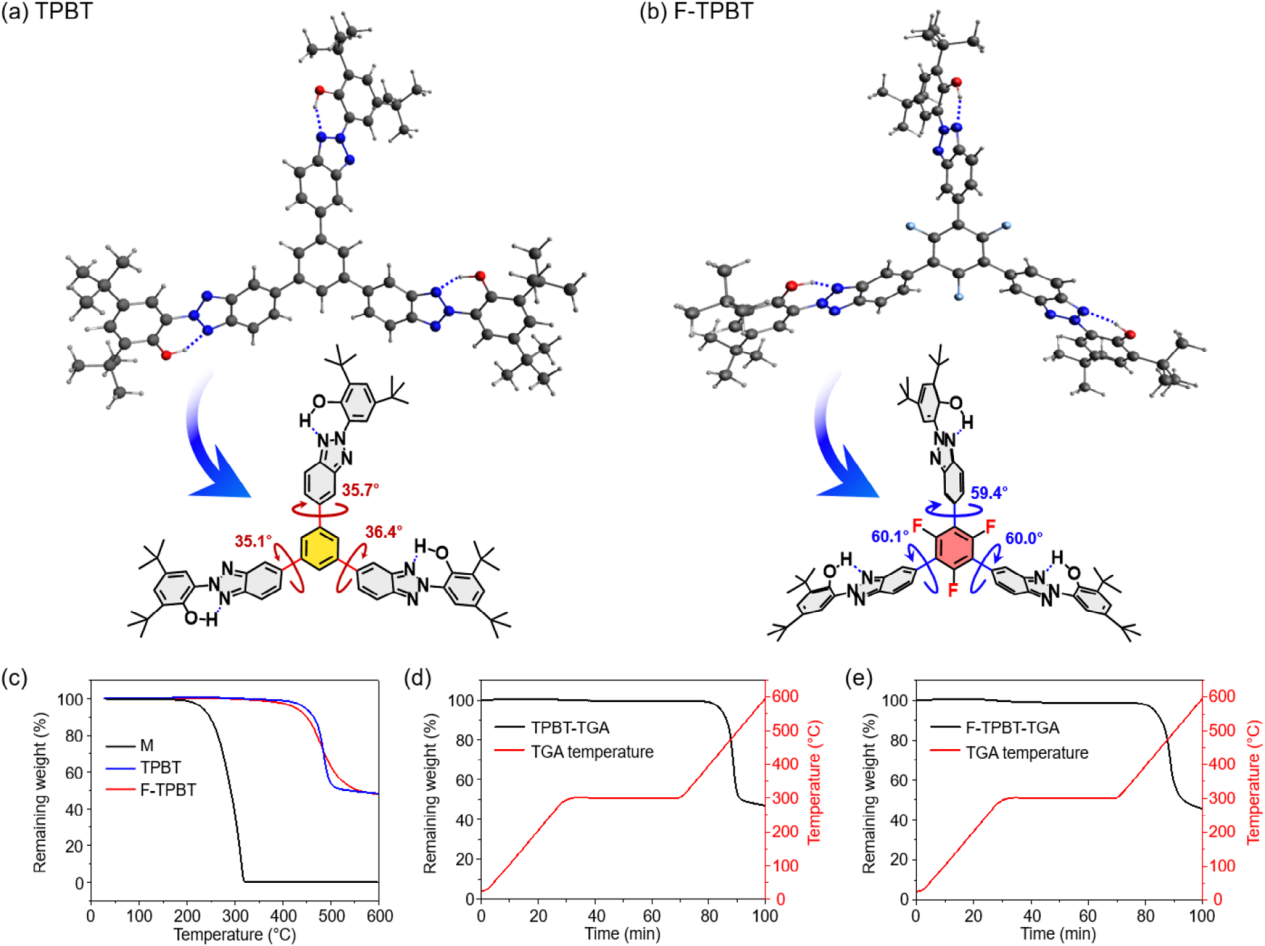
a) Calculated optimized structure of TPBT; b) calculated optimized structure of F‐TPBT; c) TGA thermogram of UV absorbers over the temperature range 25–600 °C at a heating rate of 10 °C/minute; d, e) TGA thermograms of TPBT and F‐TPBT, respectively, measured over 25–600 °C with an isothermal hold of 40 minutes at 300 °C and a heating rate of 10 °C/minute.

The optimized structures of TPBT (Figure 2a) and F‐TPBT (Figure 2b) were obtained through DFT calculations (see Supporting Information for details).^[^ ^24^ ^]^ The dihedral angles between the central phenyl linker and substituted M units in TPBT (35–36°) were lower than those in F‐TPBT (59–60°), as expected. The higher angles in F‐TPBT reflected the steric hindrance induced by the fluorines in the linker, which increased the distortion of the substituted M units.

To correlate the calculated structures with thermal stability, we subjected M, TPBT, and F‐TPBT to TGA, which was performed by heating from 25 to 600 °C under N_2_ at 10 °C/minute (Figure 2c, Table 1). M rapidly decomposed below 312 °C, whereas TPBT was highly thermally stable due to enhanced intermolecular interactions (as reported in our previous studies), showing 1, 5, and 10 wt% decomposition temperatures of 400, 445, and 464 °C, respectively. F‐TPBT exhibited a minimally lower thermal stability, showing 1, 5, and 10 wt% decomposition temperatures of 361, 427, and 449 °C, respectively. Additionally, we subjected TPBT and F‐TPBT to isothermal TGA at 300 °C/40 minute (Figure 2d,e, respectively; Table 1). At the end of the isothermal analysis, the mass losses were only 0.11 and 0.32 wt%, respectively, indicating that the compounds are highly thermally stable. Thus, trimerization effectively enhanced intermolecular interactions and increased thermal stability.

**Table 1:** Parameters of UV absorber decomposition extracted from TGA curves recorded under N_2_ within the 25–600 °C range at a heating rate of 10 °C/minute. The analysis included a 40 minutes hold at 300 °C.

	M	TPBT	F‐TPBT
1 wt% loss (°C)	197	400	361
5 wt% loss (°C)	230	445	427
10 wt% loss (°C)	247	464	449
wt% loss under isothermal conditions	‐	0.11	0.32

**Figure 3:**
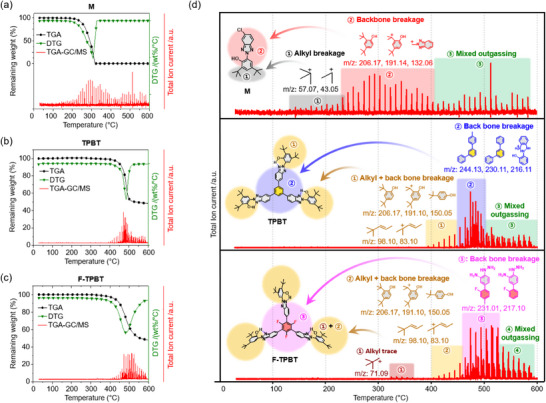
a–c) Overview of TGA‐GC/MS results correlated with TGA and DTG curves, and d) detailed TGA‐GC/MS analysis of UV absorbers over a temperature range of 25–600 °C at a heating rate of 10 °C/minute in a He gas atmosphere.

The three UV absorbers were subjected to TGA‐GC/MS, which involved heating under He gas atmosphere from 25 to 600 °C at a rate of 10 °C/minute (Figure 3). The gaseous decomposition products were injected into the GC/MS system at 1 minute intervals, and their molecular masses were monitored in real time. Figure 3 presents the correlated TGA, differential thermogravimetry (DTG), and TGA‐GC/MS results for the UV absorbers. The thermal decomposition rates ((*dm*/*dT*)_max_ (wt%/°C)) of M, TPBT, and F‐TPBT were maximal at 313, 485, and 484 °C, respectively (Figure 3(a–c), DTG curves). M exhibited continuous outgassing from 200 to 600 °C due to thermal decomposition, whereas the thermal decomposition of TPBT and F‐TPBT occurred at notably higher temperatures. Figure 3d provides a more detailed comparison of the TGA‐GC/MS results, revealing that M exhibited first outgassing peaks at *m/z* 43.05 and 57.07 around 150 °C. This result was attributed to the cleavage of thermally labile alkyl groups.^[^ ^25^ ^]^ The major outgassing peaks at *m/z* 132.06, 191.14, and 206.17 detected above 220 °C reflected the decomposition of the HBT backbone. Above 400 °C, peaks with random *m/z* values were observed.

Unlike M, TPBT, and F‐TPBT were stable below 400 °C, showing no notable thermal decomposition of alkyl groups. Above 380 °C, major peaks at *m/z* 83.10, 98.10, 150.05, 191.10, and 206.17 were observed, suggesting the simultaneous degradation of the backbone and alkyl groups. Additionally, TPBT exhibited backbone decomposition within the 450–510 °C range, whereas F‐TPBT showed a broader decomposition range of 450–530 °C. The extended decomposition temperature range of F‐TPBT was ascribed to the high dissociation energy of the C–F bond (526 J/mol) in the linker.^[^ ^26^ ^]^ F‐TPBT exhibited weak outgassing at *m/z* 71.09 within the 330–360 °C range, which was ascribed to the decomposition of an alkyl impurity. This observation aligns with the 1, 5, and 10 wt% thermal decomposition temperatures of F‐TPBT being minimally lower than those of TPBT.

Based on the above, the markedly increased thermal stabilities of TPBT and F‐TPBT were mainly due to their enhanced intermolecular interactions. These interactions significantly increased the thermal decomposition temperatures of both the alkyl groups and the molecular backbone.

**Figure 4:**
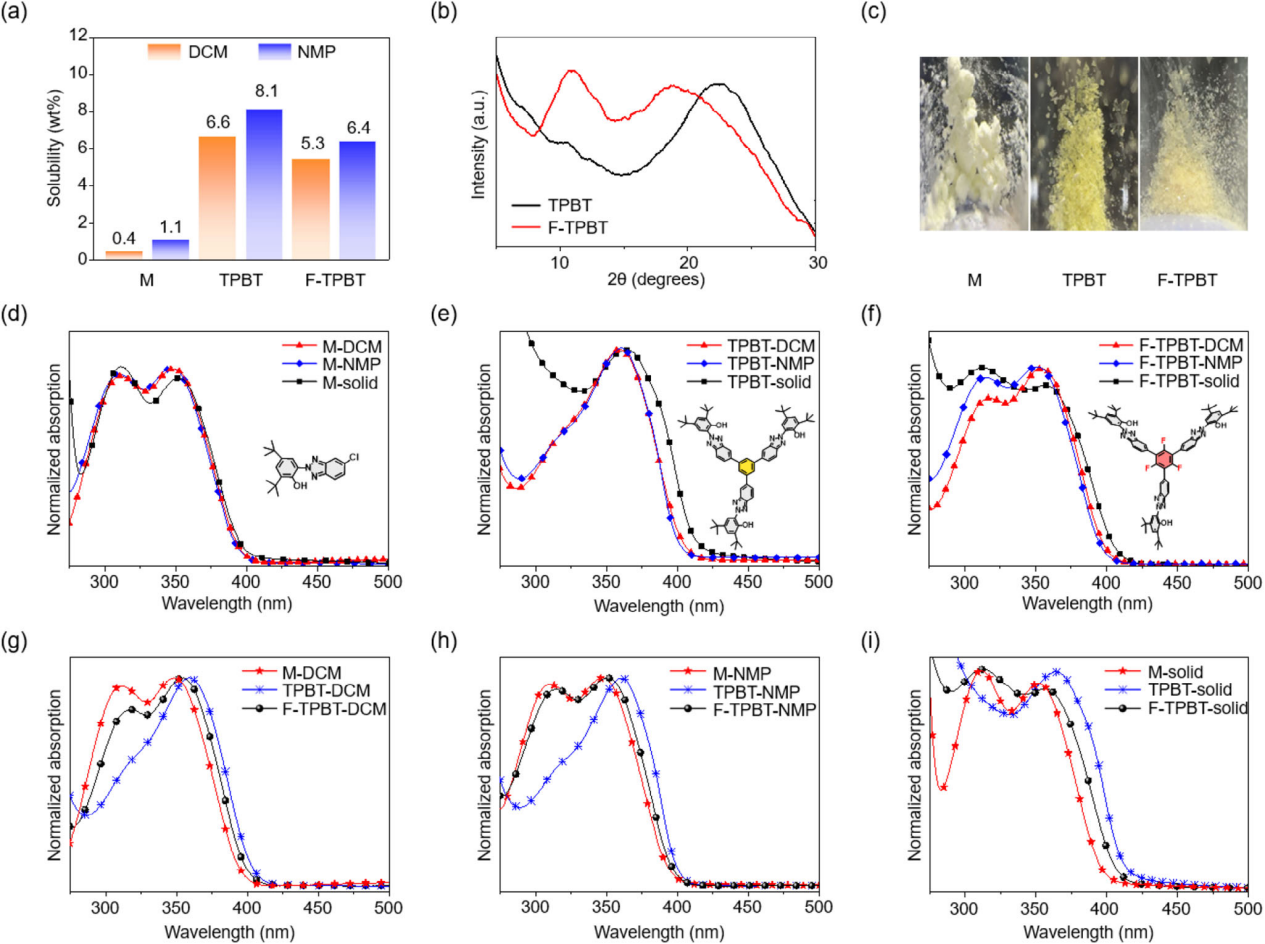
a) Solubility in DCM and NMP; b) powder XRD pattern; c) powder image; and d–i) optical absorption spectra of M, TPBT, and F‐TPBT.

The structure‐correlated optical properties of the UV absorbers were probed by solubility measurements in dichloromethane (DCM) and NMP (Figure 4a), powder XRD analysis (Figure 4b), powder imaging (Figure 4c), and UV‐visible absorption spectroscopy (Figure 4d–i). The parameters extracted from the UV–visible spectra are listed in Table 2. M exhibited solubilities of 0.4 and 1.1 wt% in DCM and NMP, respectively. TPBT and F‐TPBT exhibited notably higher solubilities (6.6 and 8.1 wt% for TPBT and 5.3 and 6.4 wt% for F‐TPBT in DCM and NMP, respectively). The enhanced trimer solubility was attributed to the structural distortion induced by the substituted monomers on the central (fluoro)phenyl core. Although this distortion could have minimally reduced the thermal stability of the trimeric structure by hindering π‐π stacking, it markedly enhanced solubility, which is an essential property for practical applications, such as thin‐film additives.

**Table 2:** Parameters of UV absorbers extracted from their UV–visible spectra.

	M	TPBT	F‐TPBT
Solution *λ*_max_ (nm)	310, 348	360	312, 351
Solid *λ*_max_ (nm)	311, 350	363	312, 354
Extinction coefficient (*ε*)	11,000	79,000	77,000

M exhibited a highly amorphous structure without strong XRD peaks. In contrast, the XRD patterns of TPBT and F‐TPBT (which were also amorphous) featured broad peaks at 2*θ* = 22° (TPBT) and 11° and 19° (F‐TPBT), indicating that the trimer structure influenced solid‐state stacking behavior.

The powders of M and F‐TPBT were white, whereas that of TPBT was yellow. The maximum absorption wavelengths (*λ*_max_) of M in the solution state were observed at 310 and 348 nm, and no solvatochromic shifts were detected (Figure 4d, Table 2). In the solid state, the *λ*_max_ values of M were observed at 312 and 350 nm. The molar extinction coefficient (*ε*) of M was 11,000. In contrast, TPBT exhibited a single peak at *λ*_max_ = 360 nm in the solution state and *λ*_max_ = 363 nm in the solid state, showing an increased *ε* of 79,000 (Figure 4e, Table 2). This result was indicative of an extended π‐conjugation due to the increased p‐orbital overlap among the substituted monomers around the linker core. F‐TPBT exhibited two strong absorption peaks at *λ*_max_ = 312 and 351 nm in the solution state and 312 and 354 nm in the solid state, featuring *ε* = 77,000 (Figure 4f, Table 2). The spectrum of F‐TPBT had a shape nearly identical to that of the spectrum of M, showing two distinct peaks. Thus, we concluded that the large distortion induced by the fluorinated core effectively suppressed p‐orbital overlap among the substituted monomers and prevented π‐extension. The above trends are visualized in the solution‐ and solid‐state spectra of the UV absorbers in Figure 4g–i. These findings indicate that the trimerization of monomers using a fluorinated linker effectively minimized spectral shape changes and red shifts, representing a promising molecular design strategy for optical materials.

**Figure 5:**
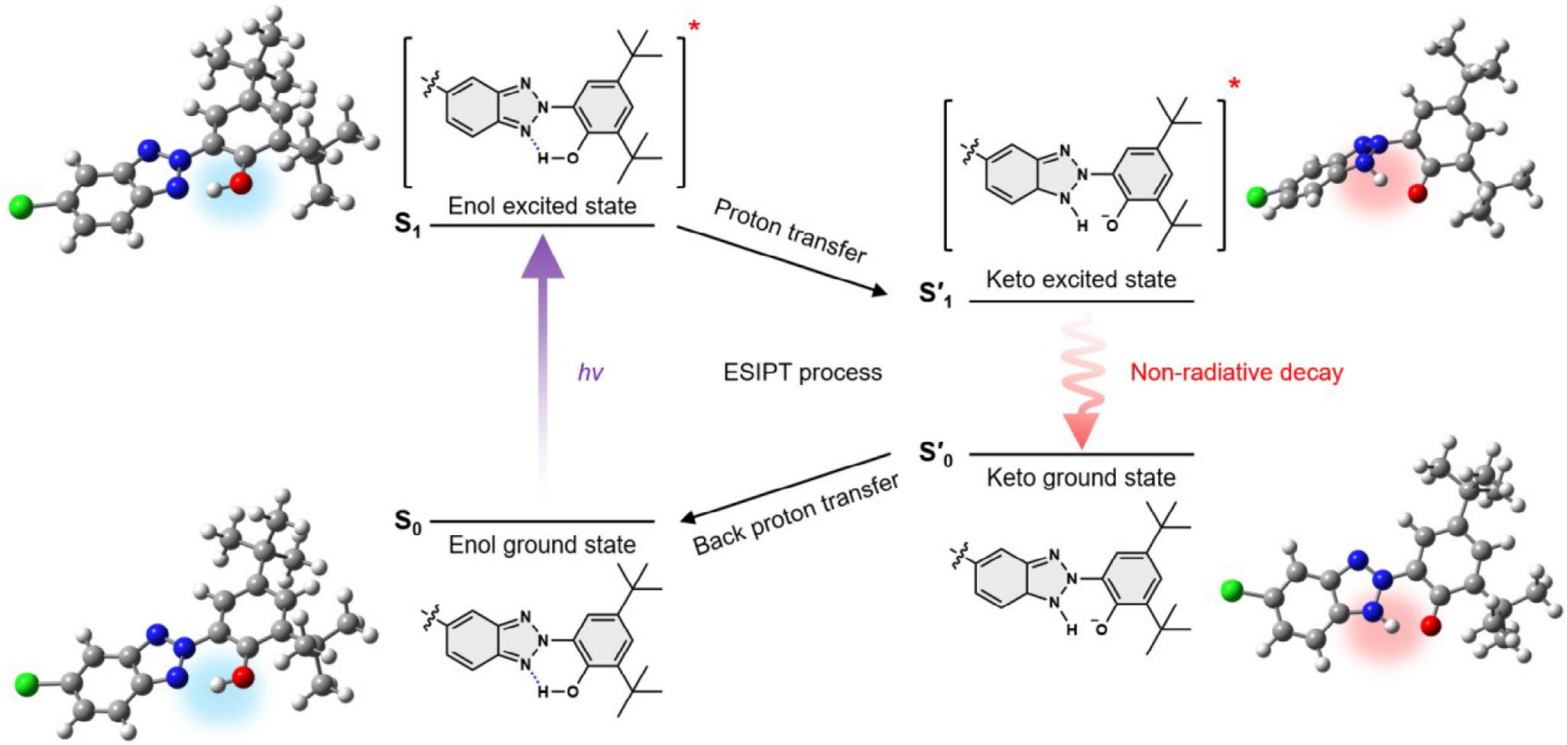
Schematic illustration of ESIPT cycle in HBT (inset: calculated optimized structures at the enol and keto states).

The ESIPT behavior of HBT was subsequently examined (Figure 5). This process originates from the presence of both proton‐donating and proton‐accepting functional groups within the molecule, facilitated by intramolecular hydrogen bonding. Upon photoexcitation, a proton transfer occurs in the excited state, yielding a photo‐tautomeric species with distinct electronic and photophysical characteristics. As a result, ESIPT‐active compounds can effectively dissipate the absorbed UV energy through nonradiative decay pathways, thereby improving their photostability and making them attractive candidates for UV‐protective applications. The inset images display the optimized geometries of the enol and keto forms in each electronic state, as determined through DFT calculations, and these results are further elaborated in the subsequent section.

**Figure 6:**
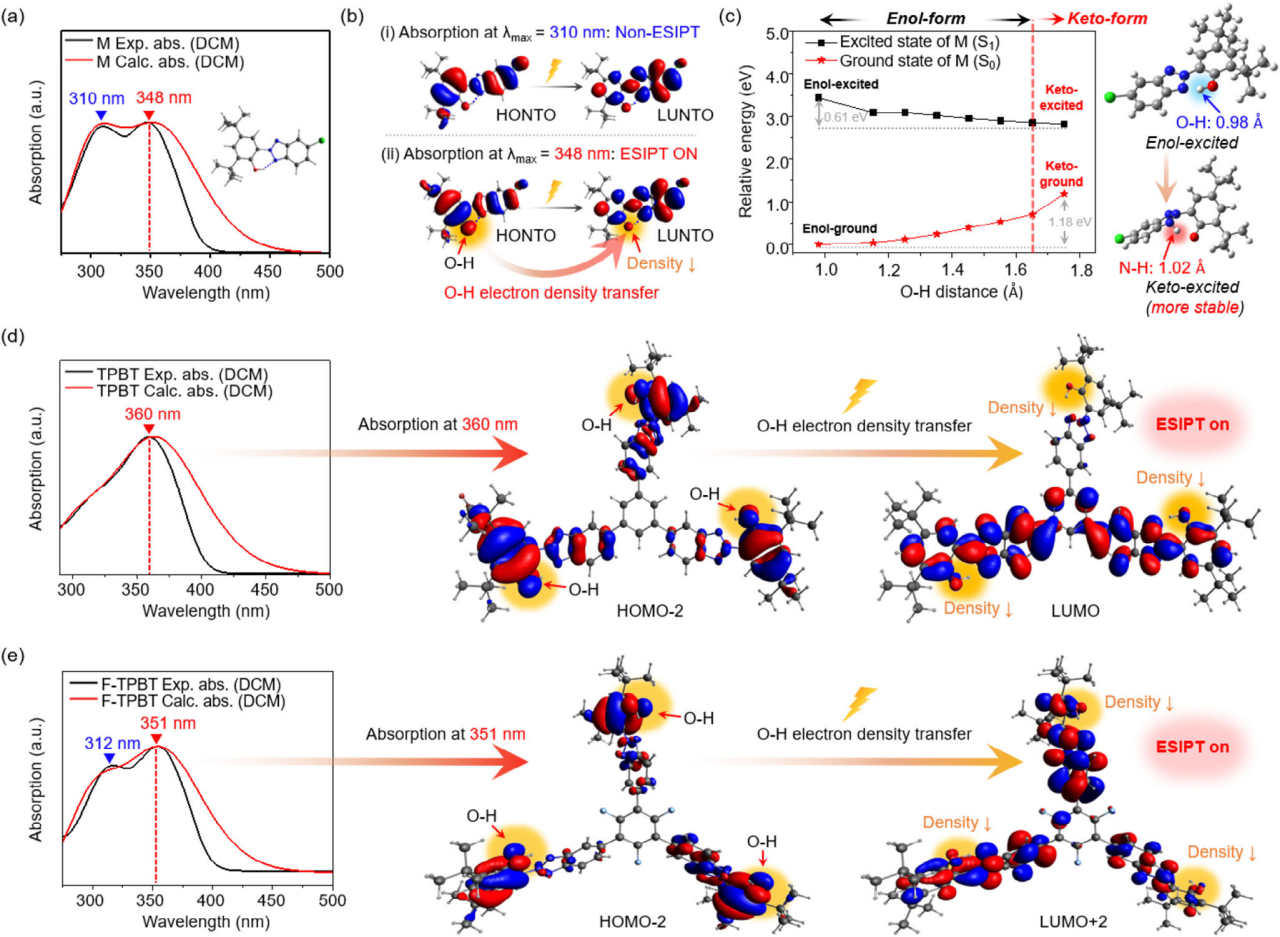
a) Calculated and experimental absorption spectrum of M (inset: optimized structure of M); b) Electron density distributions of the frontier molecular orbitals (FMOs) at *λ*_max_ of M; c) Calculated potential energy surface scan curves for the S₀ and S₁ states of M as a function of the O–H distance (inset: calculated optimized structures at the enol and keto states); d, e) Calculated and experimental absorption spectra of TPBT and F‐TPBT, respectively, (insets: electron density distributions of the FMOs corresponding to each ESIPT ON absorptions).

To confirm the occurrence of the ESIPT behavior in the actual molecule, we examined the experimental and calculated absorption spectra of the UV absorbers in DCM (Figure 6). The employed time‐dependent DFT calculations are described in the Supporting Information.^[^ ^27^ ^]^ The calculated UV‐visible spectrum of M matched well the experimental spectrum (Figure 6a). Therefore, the calculation results for M were used to interpret its experimental spectrum and electron density distribution. The electron density distribution of the FMOs at the two absorption peak wavelengths of M (Figure 6b) indicated no notable electron density redistribution between the highest occupied natural transition orbital (HONTO) and lowest unoccupied natural transition orbital (LUNTO) around the hydroxy group of M during the electronic transition at *λ*_max_ = 310 nm. However, for the LUNTO, the electron density around the hydroxy group of M during the electronic transition at *λ*_max_ = 348 nm was notably lower than that for the HONTO. The electron density change of M at *λ*_max_ = 348 nm provided the driving force for the proton in the hydroxy group (O–H, proton donor), initially present in the enol form in the ground state, to be transferred to the nitrogen atom (N–H, proton acceptor) via excited‐state intramolecular proton transfer (ESIPT). To further examine this phenomenon, we explored the potential energy surface scan curves of the excited (S_1_) and ground (S_0_) states of M as functions of the O–H bond length (Figure 6c).^[^ ^28^ ^]^ The O–H bond length in the optimized ground‐state structure of M was 0.98 Å. As the O–H bond length increased, the distance to the proton acceptor (N–H) decreased, indicating a gradual transition to the keto form. The result obtained for S_1_ revealed that the keto form is energetically more stable than the enol form by at least 0.61 eV, suggesting the conversion of M from the enol to keto form during photoexcitation. Subsequently, the keto form of M in the S_1_ state undergoes vibrational relaxation to form the S_0_ state of the keto form. However, the S_0_ state of the keto form is at least 1.18 eV higher in energy than the S_0_ state of the enol form, which indicates that the keto form is unstable in the ground state. This result suggests the occurrence of barrierless backward ESIPT, which affords the enol form of M in the S_0_ state.^[^ ^29^ ^]^

Given that M has an HBT backbone, ESIPT should also occur in TPBT and F‐TPBT, as they also have HBT backbones. Figure 6d presents the experimental and calculated UV–visible absorption spectra of TPBT, with the inset showing the electron density distribution of the FMO at *λ*_max_ = 360 nm. The absorption peak of TPBT at *λ*_max_ = 360 nm corresponds to a transition from the highest occupied molecular orbital (HOMO)‐2 to the lowest unoccupied molecular orbital (LUMO) with an oscillator strength of 1.55, indicating the strongest electronic transition. Additionally, for the LUMO, the electron density around the hydroxy group is notably lower than that of the HOMO‐2, which suggests that this transition is accompanied by ESIPT. The high electron density around the linker observed for the LUMO suggests π‐extension resulting from the increased π‐orbital overlap among the substituted monomers. Figure 6e presents the experimental and calculated UV–visible absorption spectra of F‐TPBT, with the inset showing the electron density distribution of the FMO at *λ*_max_ = 351 nm. The absorption peak at *λ*_max_ = 351 nm corresponds to a transition from the HOMO‐2 to the LUMO + 2 with an oscillator strength of 1.34, indicating the strongest electronic transition. Similar to M and TPBT, the electron density in the hydroxy group region for the LUMO + 2 is notably lower than that for the HOMO‐2, confirming that ESIPT also occurs at this wavelength. The absence of considerable electron density redistribution around the hydroxy group at *λ*_max_ = 312 nm suggests that the corresponding transition is not accompanied by ESIPT. The substantially decreased electron density around the fluorinated linker observed for the LUMO + 2 of F‐TPBT suggests that the large distortion between the substituted monomers effectively suppresses π‐orbital overlap and thereby prevents π‐extension.

The above results indicate that ESIPT occurs in all three molecules (M, TPBT, and F‐TPBT) because of their HBT backbone. However, only F‐TPBT exhibits π‐extension reduction (due to the increased steric hindrance caused by the central fluorinated phenyl linker and monomers) and minimized spectral red shifts.

**Figure 7:**
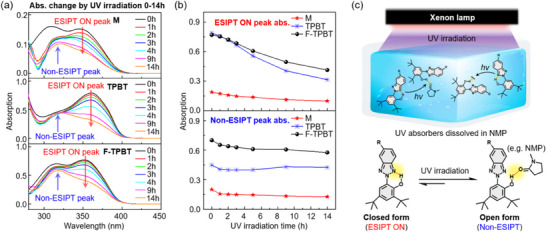
a) Absorption changes of UV absorbers in NMP solution under UV irradiation for 0–14 hours over the wavelength range of 280–450 nm; b) Absorption changes of UV absorbers at the ESIPT ON and Non‐ESIPT peaks; c) Proposed photochemical reactions illustrating the conversion from the closed form (ESIPT ON) to the open form (Non‐ESIPT) of UV absorbers induced by UV irradiation.

Subsequently, we monitored the solution‐state (10^−5^ M in NMP) UV–visible spectra of the three compounds during 14 hours UV irradiation using a 450 W/m^2^ xenon lamp as the UV light source. The spectra of all compounds in the wavelength range of 280–450 nm changed with the increasing irradiation time (Figure 7a). The absorption of M at *λ*_max_ = 348 nm corresponds to the closed form (ESIPT ON), whereas that at *λ*_max_ = 310 nm represents the open form (non‐ESIPT).^[^ ^30^ ^]^ Similarly, the absorption of F‐TPBT at *λ*_max_ = 351 nm corresponds to the ESIPT ON state, while that at *λ*_max_ = 312 nm represents the non‐ESIPT state. The absorption of TPBT at *λ*_max_ = 360 nm corresponds to the ESIPT ON state, while the new absorption peak at *λ*_max_ = 310 nm that emerges during UV irradiation represents the non‐ESIPT state. Subsequently, we examined the absorbance changes at the ESIPT ON and non‐ESIPT peak wavelengths (Figure 7b). The ESIPT ON absorption peak of M continuously lost intensity during irradiation, with the corresponding absorbance decreasing from 0.19 to 0.09. The non‐ESIPT absorbance of M initially decreased from 0.20 to 0.13. Similarly, the ESIPT ON absorption of TPBT decreased from 0.79 to 0.31 during 14 hours of irradiation, while the non‐ESIPT peak of TPBT, which was initially absent, emerged after UV exposure. Notably, F‐TPBT exhibited both ESIPT ON and non‐ESIPT peaks, maintaining the highest absorbance among the UV absorbers even after 14 hours of irradiation.

Figure 7c illustrates the photochemical reactions potentially responsible for the transition from the closed‐form ESIPT ON state to the open‐form non‐ESIPT state upon UV irradiation. The gradual decrease in the ESIPT ON absorption suggests that the absorbed UV light is stabilized through repeated forward and backward ESIPT cycles during irradiation (Figure 7a,b). However, instead of the full restoration of the original ESIPT ON state, some protons interact with the surrounding solvent molecules (e.g., NMP) or form hydrogen bonds with other molecules to form the non‐ESIPT state (Figure 7c). Although the complete recovery of the ESIPT ON state from the non‐ESIPT state was not achieved in any case, previous studies have reported that certain HBT derivatives can be molecularly designed to enable reversible ESIPT transitions.^[^ ^30^ ^]^ Moreover, although the photodegradation of the UV absorbers could contribute to the observed absorption decreasing, HBT derivatives are known for their excellent photostability. Thus, considerable photodegradation within the observed UV irradiation time is unlikely.

**Figure 8:**
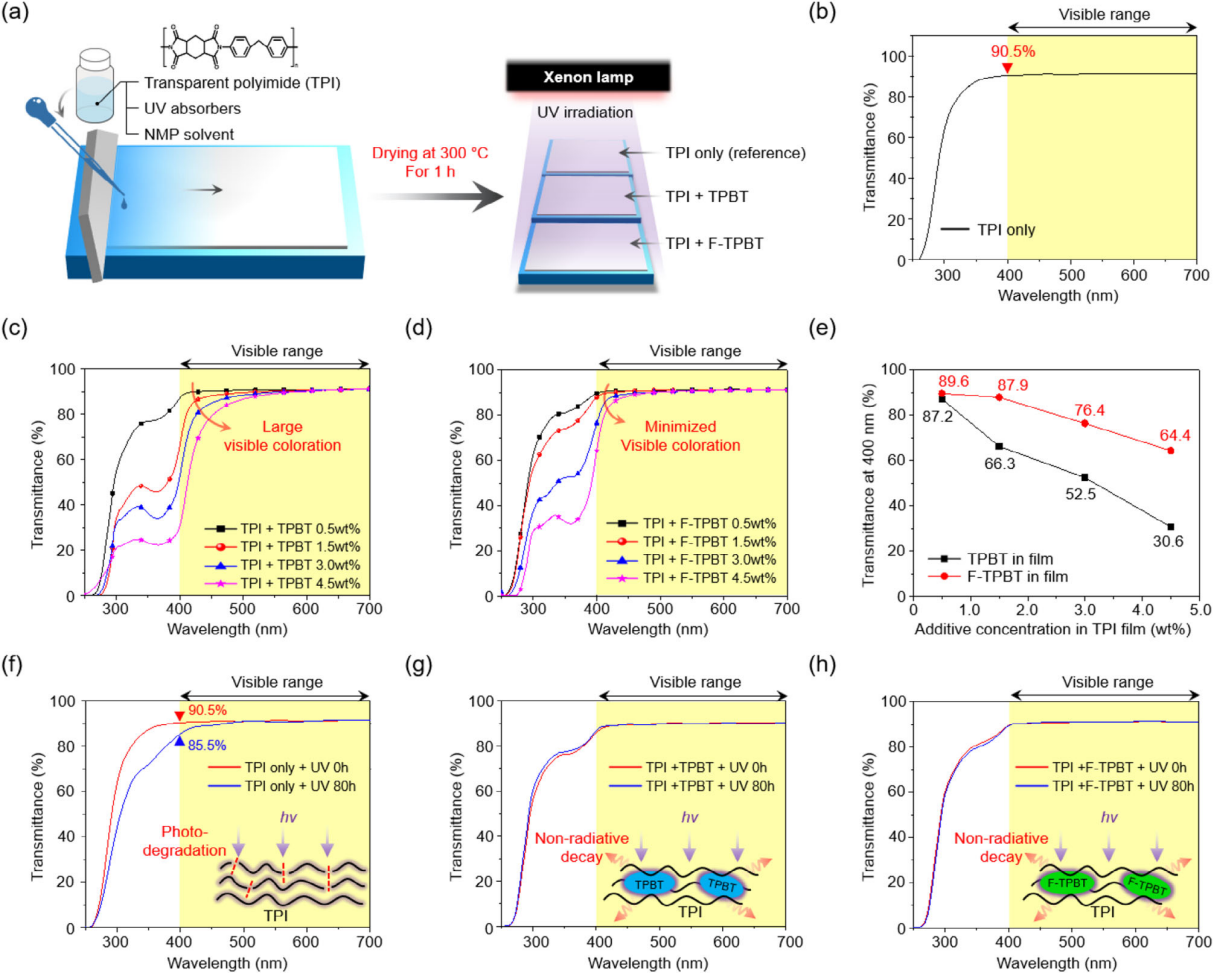
a) Schematic illustration of the TPI film fabrication process. Transmittance spectra of TPI films in the wavelength range of 250–700 nm: b) without a UV absorber, c) with TPBT at concentrations of 0–4.5 wt%, and d) with F‐TPBT at concentrations of 0–4.5 wt%. e) Transmittance at 400 nm as a function of TPBT and F‐TPBT concentrations (0–4.5 wt%). Transmittance spectra of TPI films before and after 80 hours of UV irradiation: f) without a UV absorber, g) with 0.5 wt% TPBT, and h) with 0.5 wt% F‐TPBT (inset: schematic illustration of UV‐irradiated TPI films with and without a UV‐absorber).

To evaluate the photodegradation resistance of the UV absorbers, we fabricated model TPI films with and without these absorbers and exposed them to UV irradiation. The syntheses of the model TPIs and corresponding films are detailed in the Supporting Information. A 450 W/m^2^ xenon lamp was used as the UV light source. Figure 8a summarizes the film fabrication process and photodegradation test procedure. A solution containing the model TPI, UV absorber, and NMP was blade‐coated onto a glass substrate to fabricate thin films, which were dried at 80 °C for 5 minutes and then annealed at 300 °C for 1 hour. The annealed films were exposed to UV irradiation for 80 hours, and their transmittance changes in the 250–700 nm range were analyzed. The UV absorber‐free TPI film exhibited a high transmittance in the visible range (400–700 nm) and 90.5% transmittance at 400 nm (Figure 8b). In contrast, the visible‐range transmittances of TPI films containing 0.5–4.5 wt% TPBT (Figure 8c) and F‐TPBT (Figure 8d) exhibited different trends. At TPBT contents of ≥ 0.5 wt%, a sharp transmittance drop at 400 nm was observed. In contrast, F‐TPBT‐containing TPI films exhibited a higher transmittance at 400 nm than TPBT‐containing films. This difference was confirmed by comparing the transmittances of films with different TPBT and F‐TPBT contents (0.5–4.5 wt%) at 400 nm (Figure 8e). At TPBT contents of 0.5–4.5 wt%, the transmittance at 400 nm ranged from 30.6% to 87.2%. This finding suggests that the incorporation of TPBT at high contents (≥0.5 wt%) can substantially reduce the visible‐range transmittance of TPI films. In contrast, at F‐TPBT contents of 0.5–4.5 wt%, the transmittance at 400 nm remained relatively high, ranging from 64.4% to 89.6%. Based on these results, TPI films with minimal transparency losses were selected to compare the photodegradation reduction performances of TPBT and F‐TPBT. Specifically, we examined the effect of 80 hours UV irradiation on the transmittances of TPI films containing 0.5 wt% TPBT or F‐TPBT in the 250–700 nm range (Figure 8f–h). The UV absorber‐free TPI film exhibited a 5% decrease in transmittance at 400 nm after 80 hours UV irradiation (Figure 8f), whereas TPI films containing 0.5 wt% TPBT (Figure 8g) or F‐TPBT (Figure 8h) exhibited no changes in their transmittance at 400 nm under identical conditions. This indicates that the UV absorbers embedded in the TPI matrix effectively dissipate the absorbed UV light through an ESIPT‐mediated nonradiative pathway, thereby preventing photo‐degradation of the TPI. Thus, both TPBT and F‐TPBT effectively reduced the photodegradation of TPI films. However, compared with TPBT, F‐TPBT allows for better optical transparency in TPI films while effectively suppressing their photodegradation. Although a variety of high‐performance UV‐shielding materials—‐such as quantum dots,^[^ ^31^ ^]^ organic lignin,^[^ ^32^ ^]^ and inorganic complexes^[^ ^33^, ^34^ ^]^—‐have been reported to effectively block UV radiation, their application at high concentrations typically leads to a decrease in transmittance within the visible wavelength range of the polymer matrix. In contrast, the F‐TPBT developed in this study can be incorporated into the polymer matrix at high concentrations without compromising visible‐light transmittance, while still providing excellent UV‐shielding performance.

## 4 Conclusion

The trimerization of an organic small molecule based on the use of a 1,3,5‐fluorinated phenyl linker was demonstrated to minimally affect its optical absorption spectrum while notably enhancing its stability and solubility. The trimer (F‐TPBT) exhibited a remarkably enhanced thermal stability, featuring a 1 wt% decomposition temperature of 427 °C (cf. 230 °C for the monomer), and a considerably higher solubility (5.3 wt% in DCM and 6.4 wt% in NMP; cf. 0.4 wt% in DCM and 1.1 wt% in NMP for the monomer). Moreover, F‐TPBT exhibited minimal red shifts (2–3 nm) in its absorption spectrum, which indicated that the π‐extension of the monomers attached to the fluorinated phenyl core was effectively suppressed. Based on these properties, we concluded that F‐TPBT holds promise as a UV absorber for TPI films, which are widely used in flexible displays and require ultrahigh‐temperature processing at >300 °C. Although we have validated our multimerization strategy only for a single model monomer, this approach can substantially enhance the stability and solubility of organic small molecules while minimizing red shifts in their optical spectra. In future studies, we plan to validate our strategy for a broader range of monomers. Furthermore, this study provides critical insights into improving the reliability of organic small molecules and facilitating their commercialization in optical applications.

## Conflict of Interest

The authors declare that they have no known competing interests

## Supporting information



Supporting Information

## Data Availability

The data that support the findings of this study are available from the corresponding author upon reasonable request.
