# Supplementary material for: Fluorinated Trimers for Enhancing the Stability and Solubility of Organic Small Molecules Without Spectral Shifts: Ultrastable Ultraviolet Absorbers for Transparent Polyimide Films
Source: Chemistry. 2025 Jul 2;31(45):e202501742. doi: 10.1002/chem.202501742 (PMC12351421; doi:10.1002/chem.202501742)
Supplement: Supplementary file 1 — Supporting Information [file CHEM-31-e202501742-s001.docx]

**Supporting Information**

Fluorinated trimers for enhancing the stability and solubility of organic small molecules without spectral shifts: Ultrastable ultraviolet absorbers for transparent polyimide films

Tae Gyu Hwang^a†*^, Suhyeon Kim^b†^, Hong Mo Kim^c^, Woo Jin Choi^b^, Hyun Kyu Lee^d^, Wan Soo Kim^b^, Jun Ho Yoon^b^, Yoo Sang Kim^b^, Dong Jun Lee^b^, Seung Yul Kim^a,e^, Sang Goo Lee^a*^, Jae Pil Kim^b*^

*^a^Interface Materials and Chemical Engineering Research Center, Korea Research Institute of Chemical Technology (KRICT), Daejeon 34114, Republic of Korea*

*^b^Laboratory of Organic Photo-functional Materials, Department of Materials Science and Engineering, Seoul National University, Seoul 08826, Republic of Korea*

*^c^Semiconductor Analysis Team, Gyeonggi-do Semiconductor Innovation Center, Advanced Institute of Convergence Technology, Yeongtong-gu, Suwon-si, Gyeonggi-do 16229, Republic of Korea*

*^d^*Material & Component Convergence R&D Department, Korea Institute of Industrial Technology (KITECH), Ansan 15588, Republic of Korea

^e^*Division of Materials of Science and Engineering, Hanyang University, 222 Wangsimni-ro, Seongdong-gu, Seoul 04763, Republic of Korea*

*Corresponding authors. Emails: [taegyu48@krict.re.kr](mailto:taegyu48@krict.re.kr) (T. G. Hwang), sgoo@krict.re.kr (S. G. Lee), and [jaepil@snu.ac.kr](mailto:jaepil@snu.ac.kr) (J. P. Kim).

^†^These authors contributed equally.

**Table of Contents**

**1. 1 General S3**

**1.2 Synthesis of compounds**

**Scheme S1.** Synthesis of M-pin **S3**

**Scheme S2.** Synthesis of TPBT **S4**

**Scheme S3**. Synthesis of F-TPBT **S4–5**

**Scheme S4.** Synthesis of PI-HD **S6**

**Figure S1.** (a) Synthesized PI-HD powder and (b) coloration of PI-HD in NMP **S7**

**1.3 Film fabrication**

**Figure S2.** Side-view scanning electron microscopy image of PI-HD film **S8**

**1.4 Characterization**

**Figure S3.** ^1^H NMR spectrum of M-pin in CD_2_Cl_2_ **S9**

**Figure S4.** ^13^C NMR spectrum of M-pin in CD_2_Cl_2_ **S9**

**Figure S5.** ^1^H NMR spectrum of TPBT in CD_2_Cl_2_ **S10**

**Figure S6.** ^13^C NMR spectrum of TPBT in CD_2_Cl_2_ **S10**

**Figure S7.** ^1^H NMR spectrum of F-TPBT in CD_2_Cl_2_ **S11**

**Figure S8.** ^13^C NMR spectrum of F-TPBT in CD_2_Cl_2_ **S11**

**Figure S9.** ^1^H NMR spectrum of PAA-HD in (CD_3_)_2_SO **S12**

**Figure S10.** ^1^H NMR spectrum of PI-HD in (CD_3_)_2_SO  **S12**

**Figure S11.** MALDI-TOF mass spectrum of M-pin **S13**

**Figure S12.** MALDI-TOF mass spectrum of TPBT **S13**

**Figure S13.** MALDI-TOF mass spectrum of F-TPBT **S14**

**Figure S14.** FT-IR spectrum of TPBT. **S14**

**Figure S15.** FT-IR spectrum of F-TPBT. **S15**

**Figure S16.** DSC curve of TPBT (2^nd^ cycle). **S15**

**Figure S17.** DSC curve of F-TPBT (2^nd^ cycle). **S15**

**Figure S18.** Gel permeation chromatography (GPC) trace of PI-HD  **S16**

**Table S1.** Parameters extracted from the GPC trace of PI-HD  **S16**

**1.5 Theoretical calculations S17**

1.1. General

Tinuvin 327 was purchased from Tokyo Chemical Industry. All chemicals were purchased from commercial suppliers and used as received without further purification. Transparent glass substrates were provided by NTP, Inc. ^1^H and ^13^C nuclear magnetic resonance (NMR) spectra were recorded on a Bruker Avance III HD instrument at 500 and 850 MHz in dichloromethane-*d_2_*. Matrix-assisted laser desorption/ionization time-of-flight (MALDI-TOF) mass spectra were recorded on an Applied Biosystems Voyager-DE STR biospectrometry workstation using cyano-4-hydroxycinnamic acid as a matrix. Thermogravimetric analysis combined with gas chromatography and mass spectrometry (TGA-GC/MS) was performed using NETZSCH TG 209 F1 Libra® (TGA) and Agilent 8890 GC/5977B MSD (GC/MS) instruments. Single TGA was performed using a TA instrument SDT Q600 instrument. Differential scanning calorimetry (DSC) was recorded on a TA instrument DSC Q1000. All samples were heated at a heating rate of 10 °C/min for TGA and DSC analysis. Ultraviolet–visible (UV–vis) absorption spectra were recorded using a SHIMADZU UV-1900 UV–vis spectrophotometer. UV irradiation test was performed using an ATLAS Suntest CPS+ instrument. Cross-sectional scanning electron microscopy (SEM) image of film sample was obtained using FEI Helios NanoLab 450.

1.2. Synthesis of 2,4-di-tert-butyl-6-(5-(4,4,5,5-tetramethyl-1,3,2-dioxaborolan-2-yl)-2H-benzo[d][1,2,3]triazol-2-yl)phenol (M-pin)


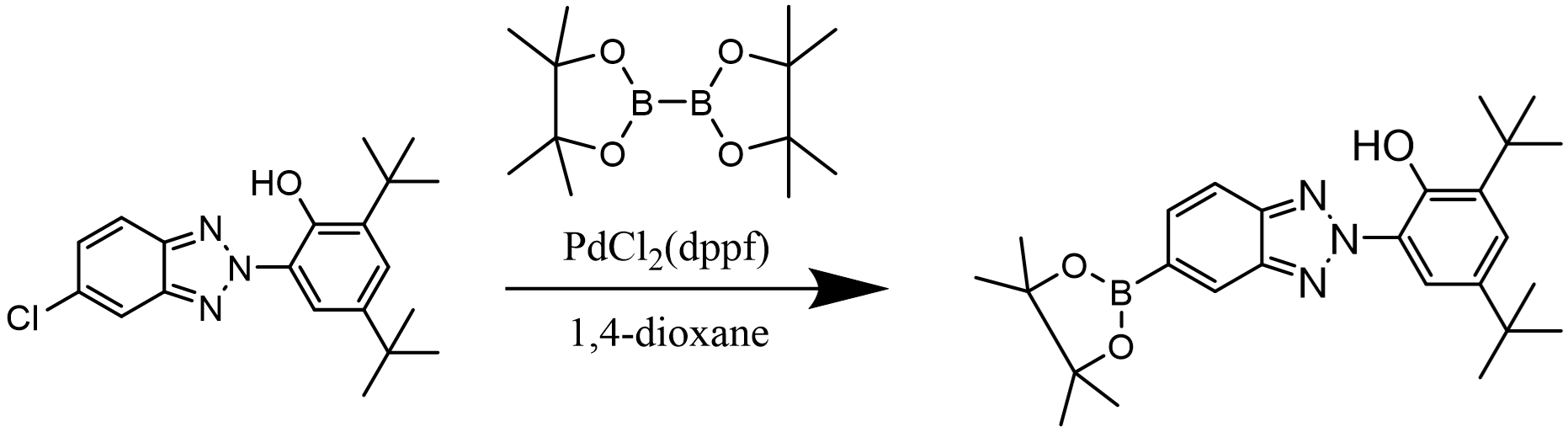


**Scheme S1.** Synthesis of M-pin.

Tinuvin 327 (3.00 g, 8.3 mmol), PdCl_2_(dppf) (0.61 g, 0.68 mmol), and 1,4-dioxane (50 mL) were added into a three-neck round-bottom flask. Then, bis(pinacolato)diboron (2.13 g, 8.3 mmol) and potassium acetate (0.83 g, 8.3 mmol) were added, and the mixture was heated at 120 °C for 24 h, cooled to room temperature, and extracted with dichloromethane and water. The collected organic layer was dried over anhydrous MgSO_4_ and concentrated by rotary evaporation. The resulting crude product was purified by column chromatography using dichloromethane:*n*-hexane (1:1, v/v) as the eluent to obtain a light yellow powder of M-pin (2.45 g, 65.68% yield). ^1^H NMR (850 MHz, CD_2_Cl_2_), δ (ppm): 11.66 (s, 1H), 8.34 (s, 1H), 8.22 (d, *J* = 2.38 Hz, 1H), 7.85–7.83 (dd, *J_1_* = 8.5 Hz and *J_2_* = 0.68 Hz, 1H), 7.75–7.74 (d, *J* = 8.5 Hz, 1H), 7.37 (d, *J* = 2.38 Hz), 1.43 (s, 12H), 1.31–1.30 (d, *J* = 6.205 Hz). ^13^C NMR (850 MHz, CD_2_Cl_2_), δ (ppm): 147.34, 144.64, 143.08, 142.37, 139.13, 132.71, 125.97, 125.90, 125.72, 117.07, 116.78, 36.15, 35.04, 31.76, 29.86, 25.26. MALDI-TOF MS: *m/z* found 450.1883 (100%, [M + H]^+^).

6,6',6''-(benzene-1,3,5-triyltris(2*H*-benzo[*d*][1,2,3]triazole-5,2-diyl))tris(2,4-di-*tert*-butylphenol) (TPBT)


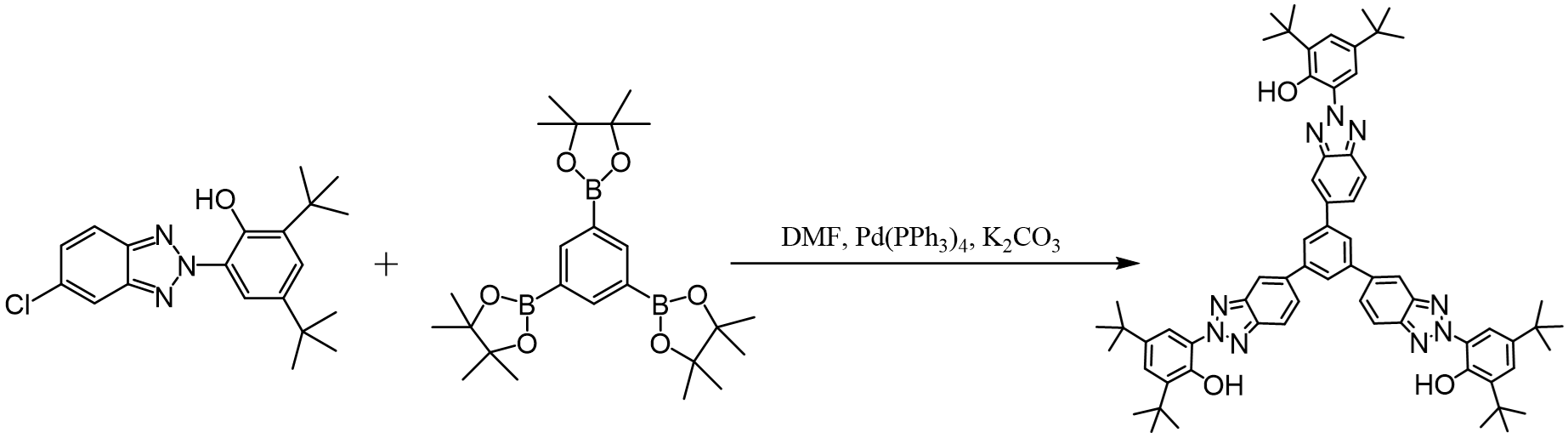


**Scheme S2.** Synthesis of TPBT.

Tinuvin 327 (1.01 g, 2.82 mmol), Pd(PPh_3_)_4_ (0.15 g, 0.13 mmol), and dimethylformamide (20 mL) were added into a three-neck round-bottom flask. The mixture was stirred at room temperature with nitrogen gas bubbling for 1 h, supplemented with 1,3,5-phenyltriboronic acid, tris(pinacol) ester (0.32 g, 0.71 mmol), and K_2_CO_3_ (64.96 mg, 0.47 mmol), refluxed for 36 h, cooled to room temperature, and extracted with dichloromethane and water. The collected organic layer was dried over anhydrous MgSO_4_ and concentrated by rotary evaporation. The crude product was purified by column chromatography using dichloromethane:petroleum ether (1:3, v/v) as the eluent to obtain light yellow crystals of TPBT (0.45 g, 61.2% yield). ^1^H NMR (500 MHz, CD_2_Cl_2_), δ (ppm): 11.74 (s, 3H), 8.32 (d, *J* = 2.5 Hz, 3H), 8.27 (s, 3H), 8.07–8.05 (d, *J* = 9.5 Hz, 3H), 8.05 (s, 3H), 7.90 (dd, *J*_1_ = 7.5 Hz and *J*_2_ = 1.5 Hz, 3H), 7.46 (d, *J* = 2.5 Hz, 3H), 1.52 (s, 27H), 1.40 (s, 27H). ^13^C NMR (125 MHz, CD_2_Cl_2_), δ (ppm): 147.32, 143.85, 142.93, 142.66, 142.42, 140.83, 139.18, 128.74, 126.76, 125.91, 125.76, 118.59, 116.72, 115.93, 36.19, 35.07, 31.80, 29.91. MALDI-TOF MS: *m/z* found 1042.5773 (100%, [M + H]^+^); C_66_H_75_N_9_O_3_ requires 1042.60 ([M + H]^+^). Melting point: 176.79 °C, FT-IR (neat): ν (cm^−1^) 3115 (intramolecular hydrogen bonding), 2954 (C-H), 1244 (C-N).

6,6',6''-((2,4,6-trifluorobenzene-1,3,5-triyl)tris(2H-benzo[d][1,2,3]triazole-5,2-diyl))tris(2,4-di-tert-butylphenol) (F-TPBT)


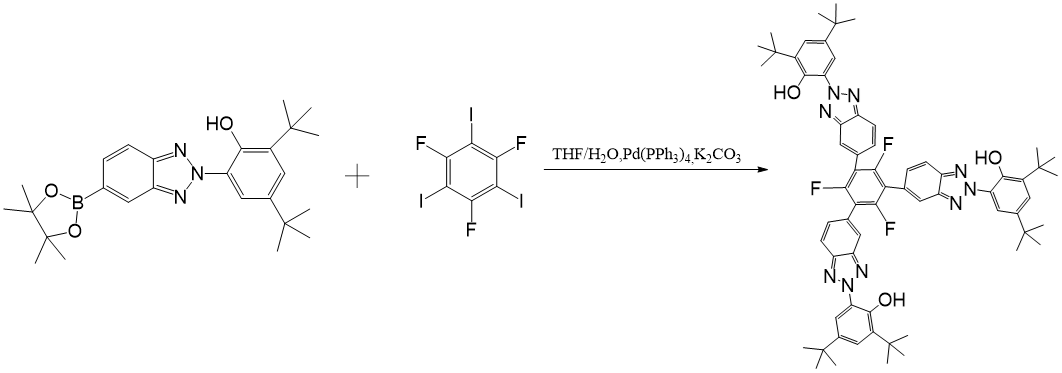


**Scheme S3.** Synthesis of F-TPBT.

1,3,5-Trifluoro-2,4,6-triiodo-benzene (0.41 g, 0.8 mmol), Pd(PPh_3_)_4_ (0.17 g, 0.15 mmol), and tetrahydrofuran (50 mL) were added into a three-neck round-bottom flask. The mixture was supplemented with M-pin (1.44 g, 3.2 mmol), K_2_CO_3_ (74.60 mg, 0.54 mmol), and distilled water (5 mL), refluxed for 24 h, cooled to room temperature, and extracted with dichloromethane and distilled water. The collected organic layer was dried over anhydrous MgSO_4_ and concentrated by rotary evaporation. The crude product was purified by column chromatography using dichloromethane:petroleum ether (1:3, v/v) as the eluent to obtain light yellow crystals of F-TPBT (0.5 g, 57.5% yield). ^1^H NMR (850 MHz, CD_2_Cl_2_), δ (ppm): 11.68 (s, 3H), 8.33 (d, *J* = 2.38 Hz, 3H), 8.20 (s, 3H), 8.11–8.10 (d, *J*_1_ = 8.925 Hz and *J*_2_ = 0.595 Hz, 3H), 7.69–7.68 (d, *J* = 8.925 Hz, 3H), 7.47 (d, *J* = 2.38 Hz, 3H), 1.52 (s, 27H), 1.40 (s, 27H).^13^C NMR (850 MHz, CD_2_Cl_2_), δ (ppm): 147.35, 143.26, 142.96, 142.48, 139.23, 130.55, 128.31, 126.08, 125.73, 120.36, 118.25, 116.81, 36.18, 35.06, 31.76, 29.87. MALDI-TOF MS: *m/z* found 1096.4760 (100%, [M + H]^+^); C_66_H_72_F_3_N_9_O_3_ requires 1096.57 ([M + H]^+^). Melting point: 162.04 °C, FT-IR (neat): ν (cm^−1^) 3132 (intramolecular hydrogen bonding), 2955 (C-H), 1245 (C-N), 1037 (C-F).

Synthesis of PI-HD


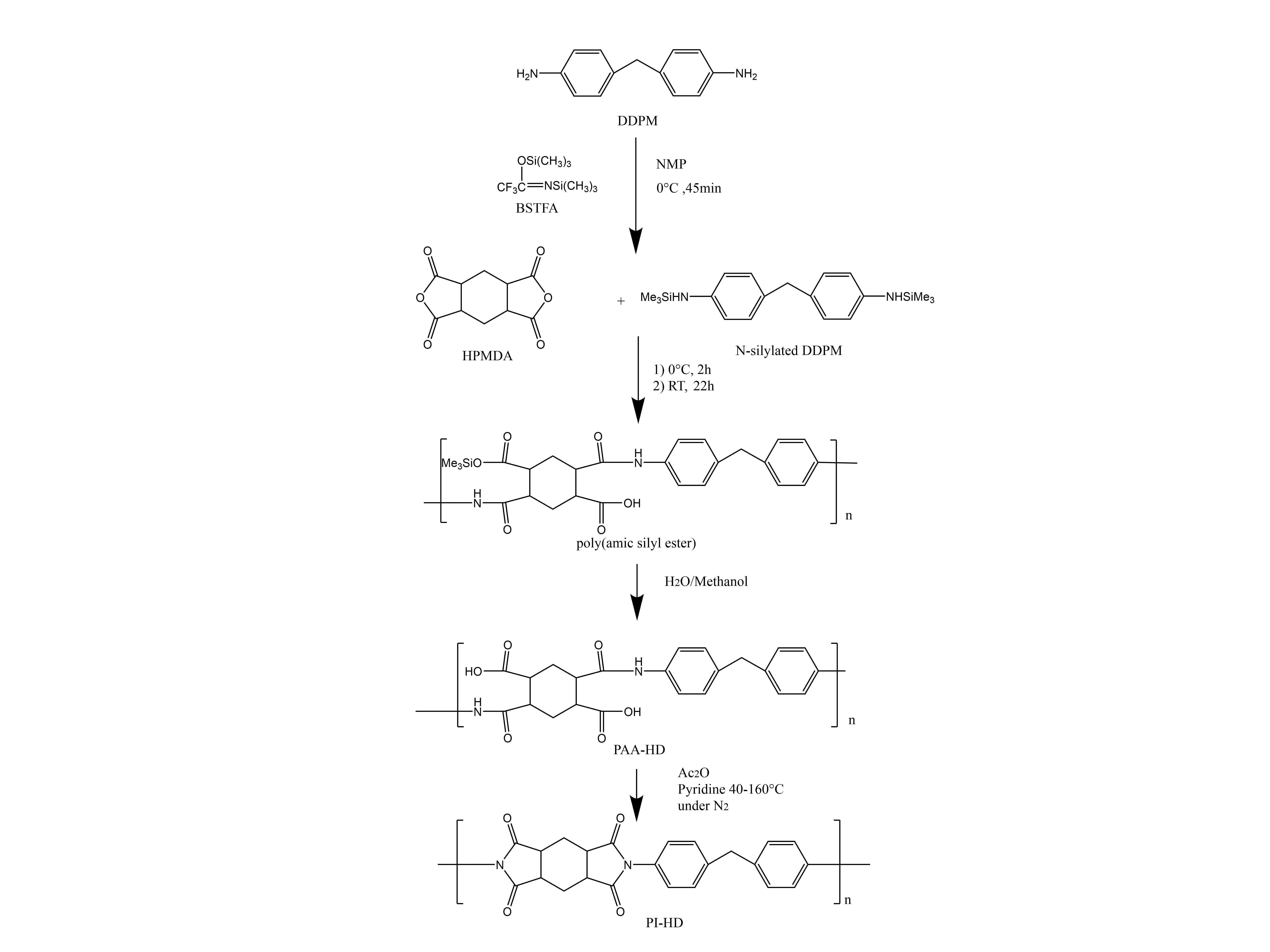


**Scheme S4.** Synthesis of PI-HD.

PI-HD was synthesized in two steps according to literature procedures: synthesis of (i) poly(amic acid) (PAA-HD) and (ii) PI-HD via the chemical imidization of PAA-HD.[1]

(i) Synthesis of PAA-HD

4,4'-Diaminodiphenylmethane (10.16 g, 0.05 mol) was dissolved in NMP (150 mL) in a 500 mL three-neck round-bottom flask under nitrogen. The solution was treated with *N*,*O*-bis(trimethylsilyl)trifluoroacetamide (2.58 g, 0.10 mol) upon stirring, stirred at 0 °C for 45 min, and then supplemented with 1,2,4,5,-cyclohexanetetracarboxylic dianhydride (11.32 g, 0.05 mol). The resultant mixture was stirred at 0 °C for 2 h and then at room temperature for 22 h, yielding a clear and viscous poly(amic acid silyl ester) solution. This solution was poured into a mixture of distilled water and methanol, and the precipitate was collected by filtration, washed with water (100 mL) and methanol (100 mL), and dried in vacuum to afford PAA-HD as a white powder (21.13 g, 93% yield). The ^1^H NMR spectrum (500 MHz, DMSO-*d*_6_) was identical to that in the literature and showed the intense signals of amide protons (N–H) at 9.44 ppm.

(ii) Synthesis of PI-HD

PAA-HD powder (3 g) was dissolved in NMP (30 mL), and the solution was stirred upon heating at 40 °C for 0.5 h. Acetic anhydride (6.8 mL) and pyridine (5.8 mL) were added for the chemical imidization of PAA-HD. The polyimide-containing mixture was cooled to room temperature and then poured into distilled water (300 mL). The precipitate was collected by filtration, washed with water (80 mL) and methanol (80 mL), and dried in vacuum to afford PI-HD as a powder (3.21 g). The number-average molecular weight (*M_n_*) and weight-average molecular weight (*M_w_*) of PI-HD were measured as 0.10 × 10^5^ and 0.13 × 10^5^ g/mol, respectively (Fig. S12 and Table S1). The imidization of PAA-HD was confirmed using ^1^H NMR spectroscopy, namely by the disappearance of the intense signals of amide protons (N–H) at 9.44 ppm observed for PAA-HD. ^1^H NMR (500 MHz, DMSO-*d*_6_), δ (ppm): 7.30–7.32 (m, ArH, 4H), 7.15–7.17 (m, ArH, 4H), 4.00 (s, 2H), 3.18 (s, 4H), 2.24–2.27 (m, 2H), 1.92–1.94 (m, 2H). The glass transition temperature (*T*_g_) of PI-HD was measured as 300 °C at a heating rate of 10 °C/min under nitrogen.


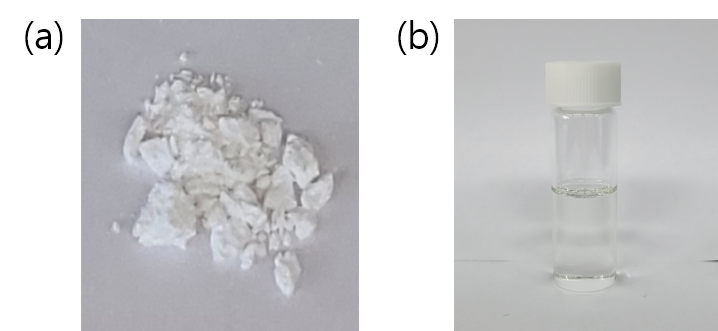


**Figure S1.** (a) Synthesized PI-HD powder and (b) coloration of PI-HD in NMP.

1.3. Film fabrication

TPBT or F-TPBT was dissolved in NMP at 0.1 mM, and the solution was supplemented with 10 wt% of PI-HD powder. The UV absorber content of the resulting solutions was 0.5–4.5 wt%. UV absorber–containing polyimide films were fabricated using the doctor blade method on transparent glass substrates. The films were prebaked at 80 °C for 5 min, and the dried films were postbaked in a convection oven at 300 °C for 1 h. The thickness of all polyimide films containing UV absorbers was ~0.5 μm. The average film thickness was analyzed by scanning electron microscopy.


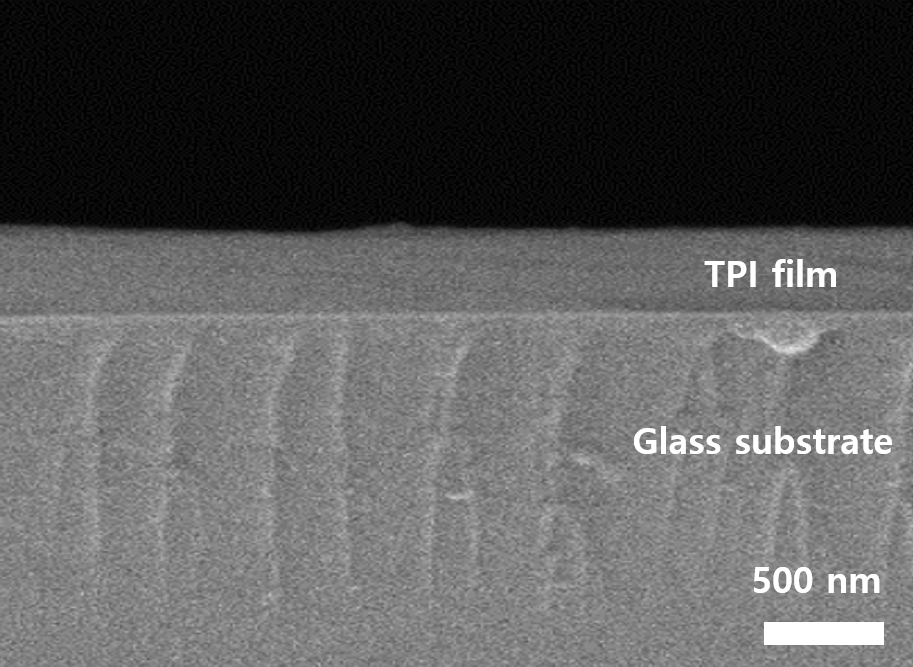


**Figure S2.** Side-view scanning electron microscopy image of PI-HD film.

1.4. Characterization


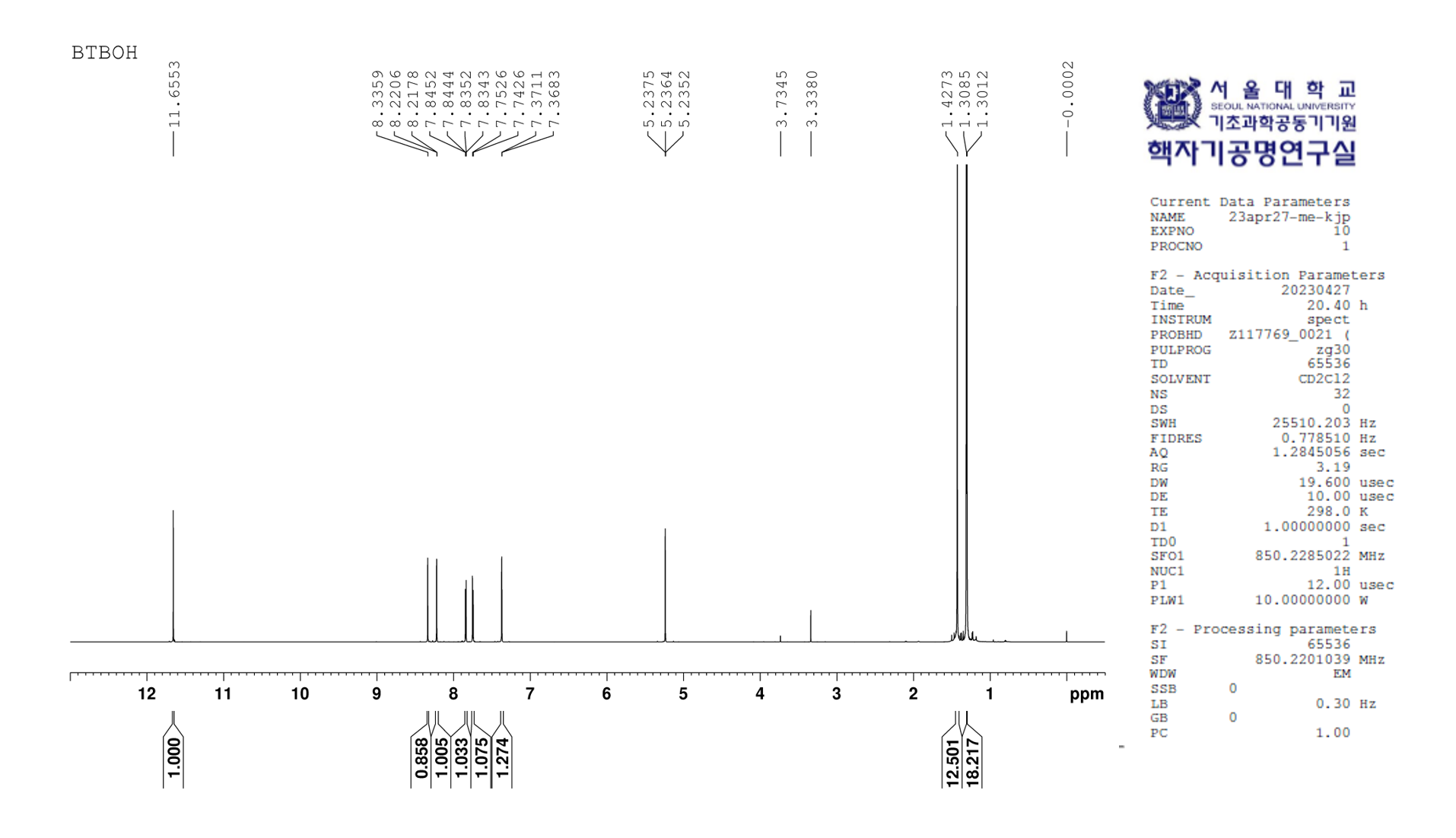


**Figure S3.** ^1^H NMR spectrum of M-pin in CD_2_Cl_2_.


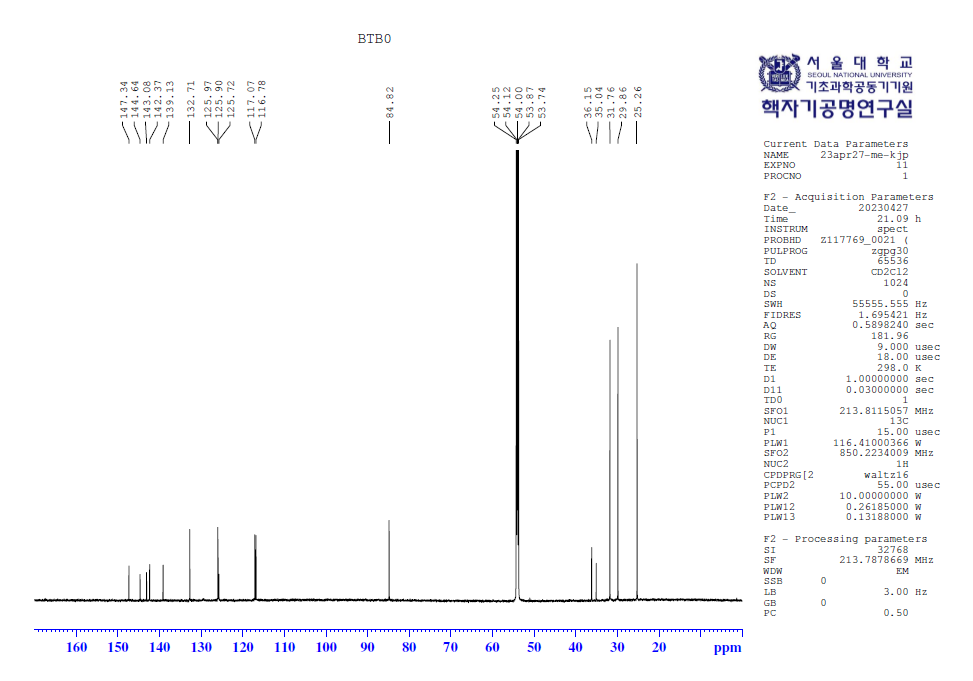


**Figure S4.** ^13^C NMR spectrum of M-pin in CD_2_Cl_2_.


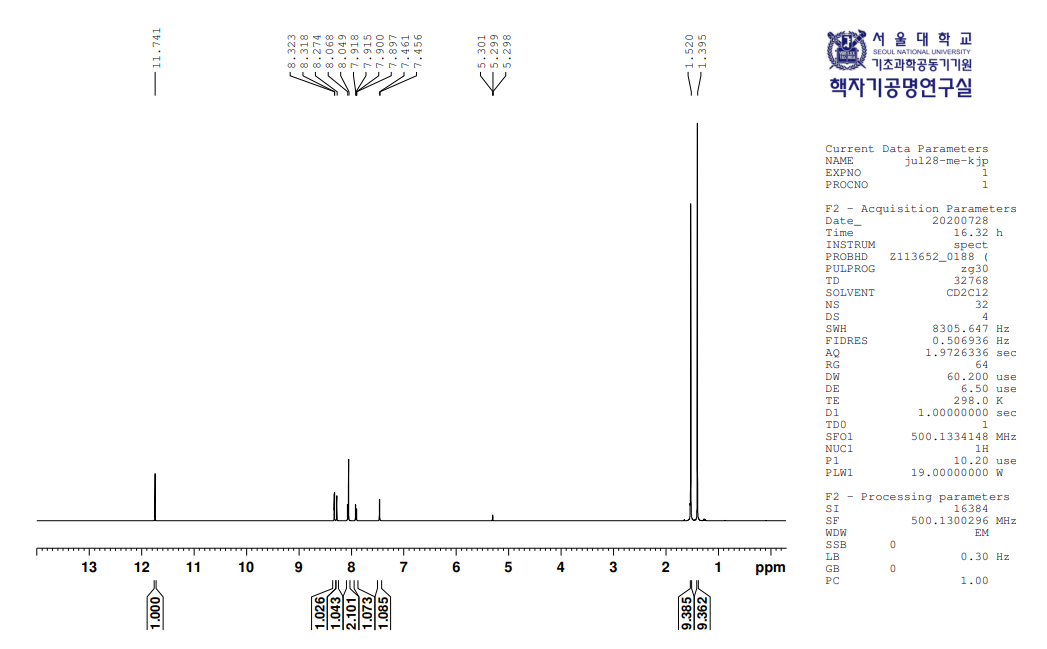


**Figure S5.** ^1^H NMR spectrum of TPBT in CD_2_Cl_2_.


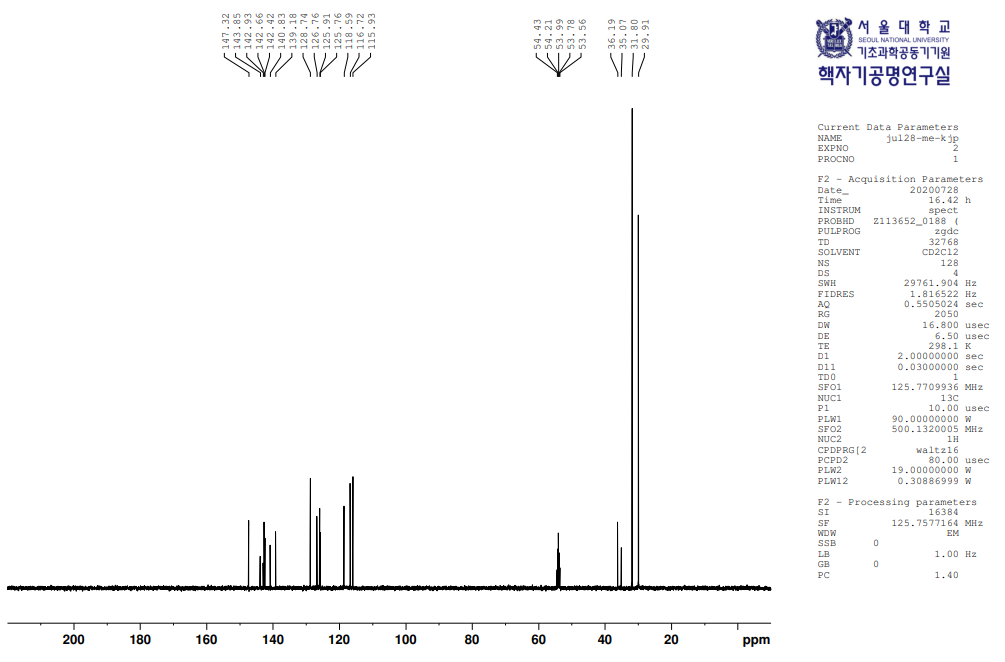


**Figure S6.** ^13^C NMR spectrum of TPBT in CD_2_Cl_2_.

**
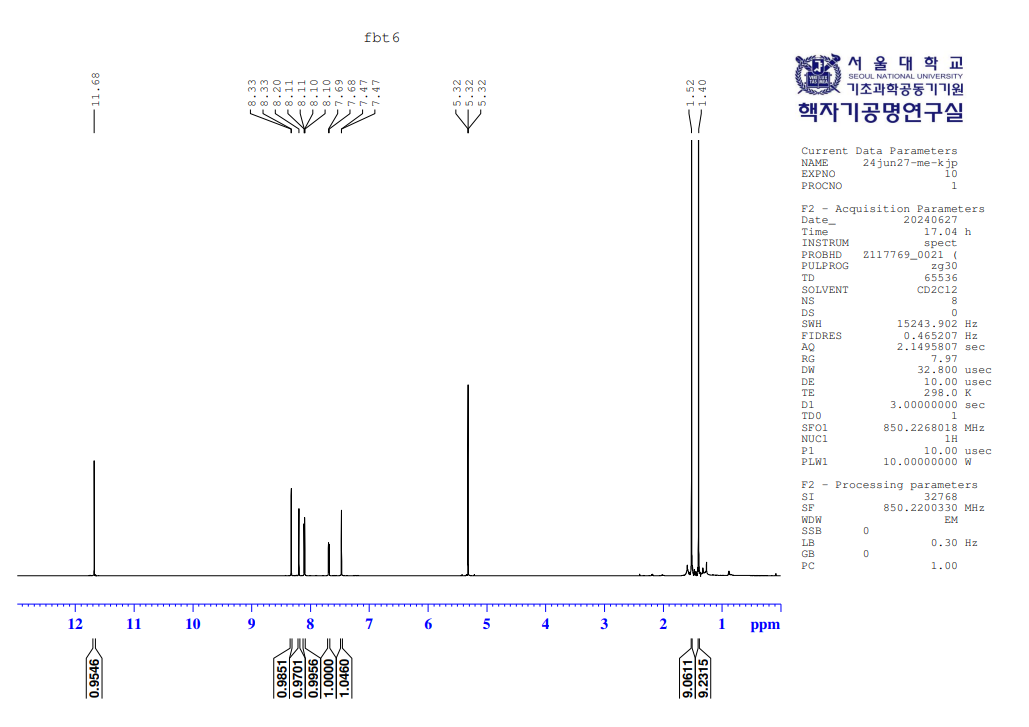
**

**Figure S7.** ^1^H NMR spectrum of F-TPBT in CD_2_Cl_2_.


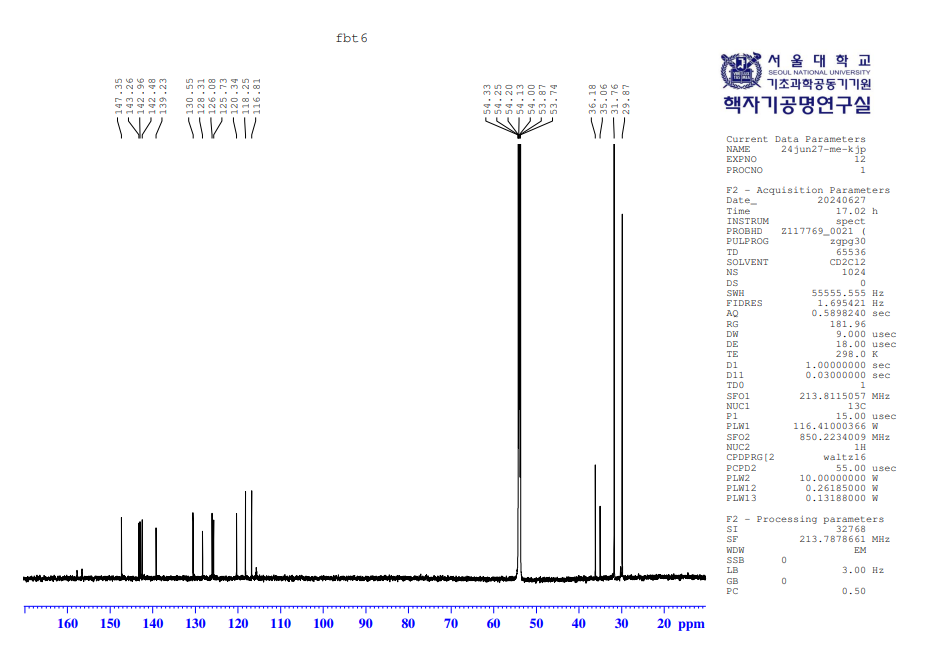


**Figure S8.** ^13^C NMR spectrum of F-TPBT in CD_2_Cl_2_.


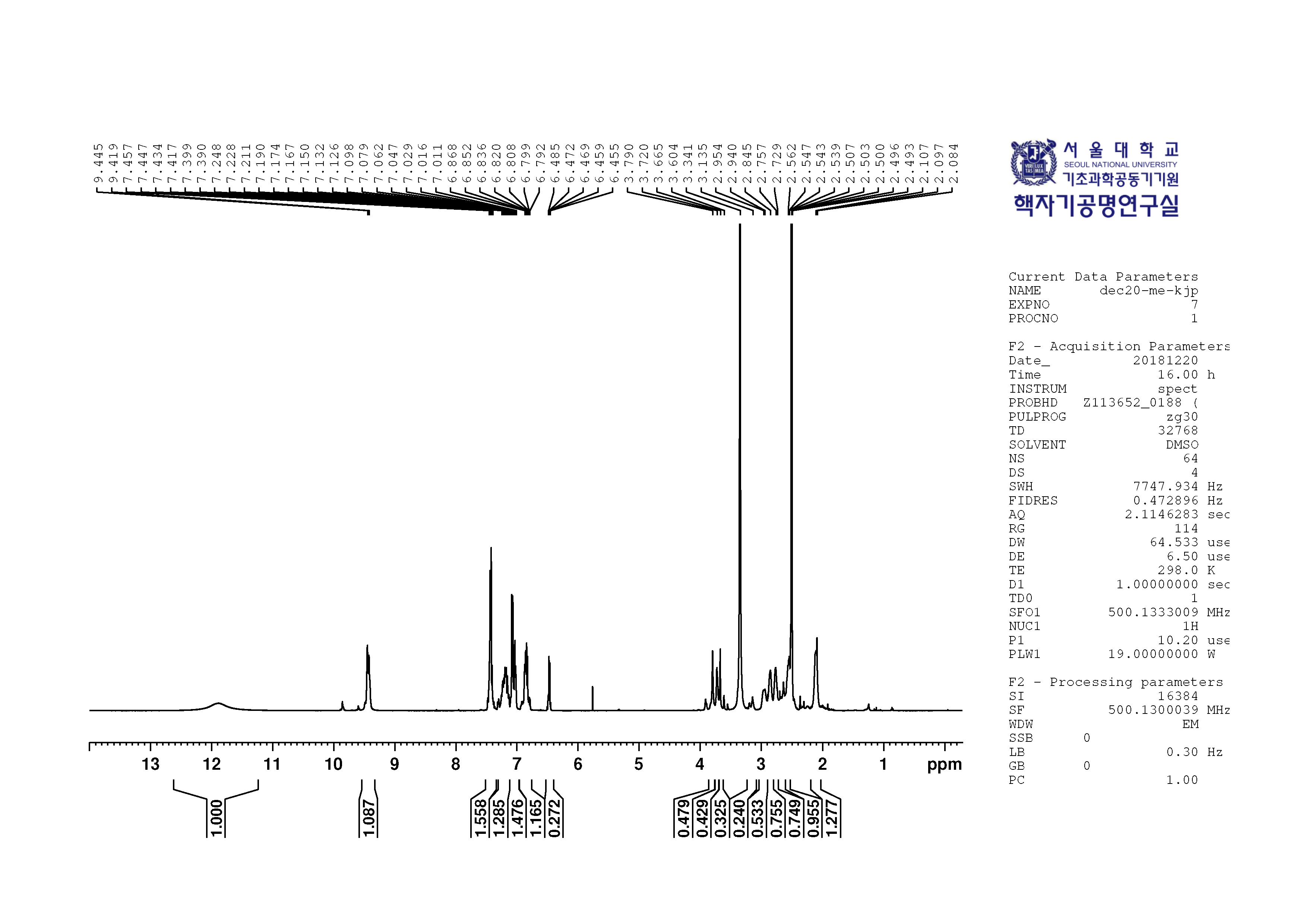


**Figure S9.** ^1^H NMR spectrum of PAA-HD in (CD_3_)_2_SO.


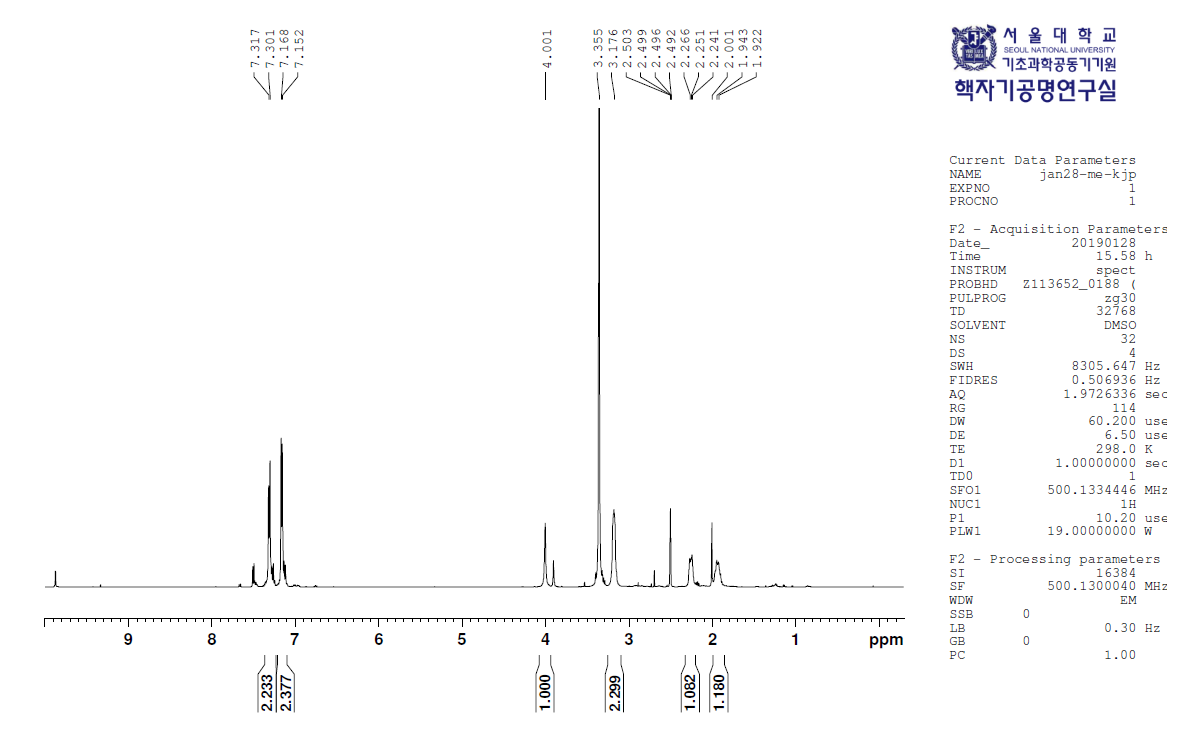


**Figure S10.** ^1^H NMR spectrum of PI-HD in (CD_3_)_2_SO.

**Figure S11.** MALDI-TOF mass spectrum of M-pin.


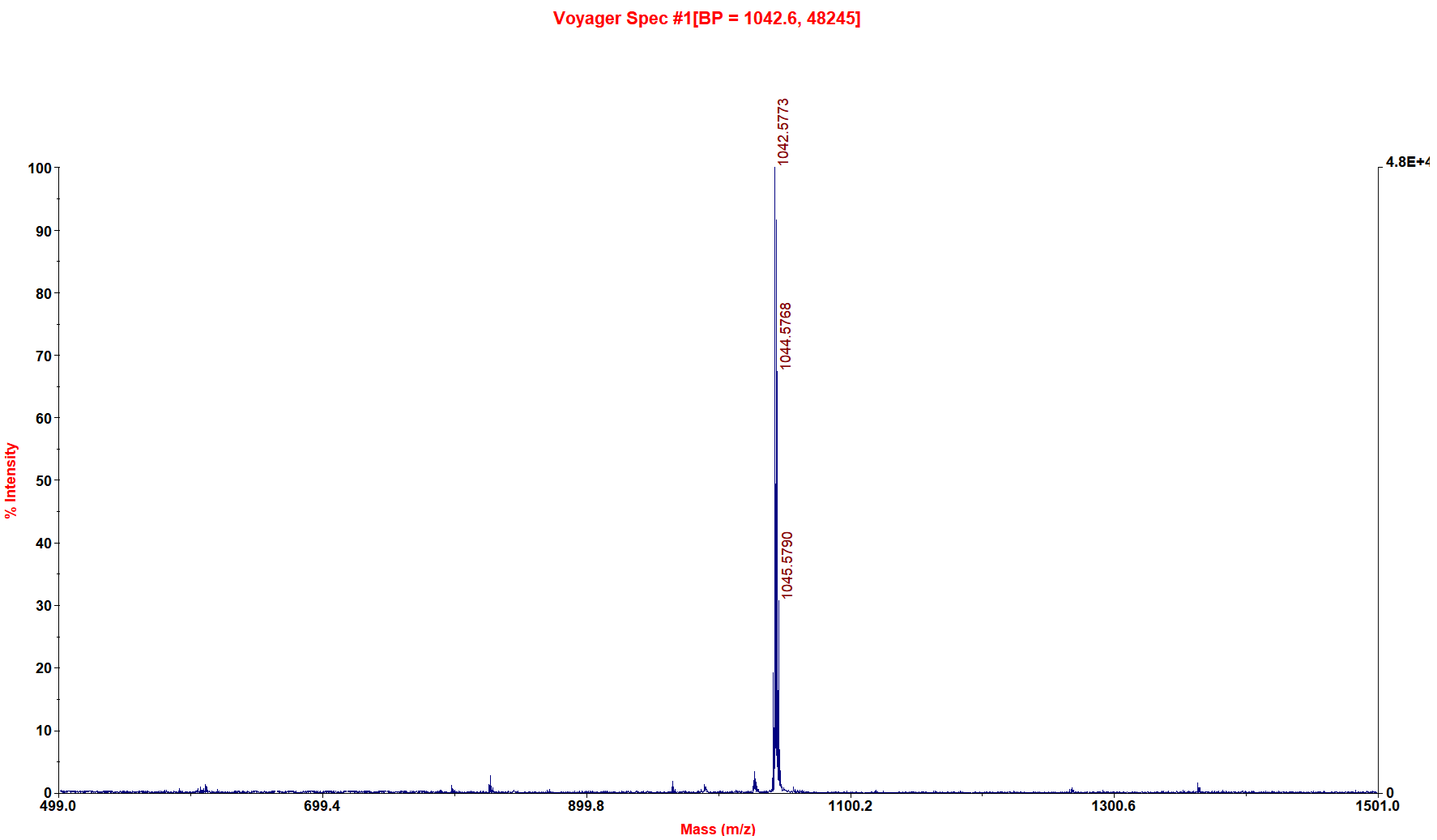


**Figure S12.** MALDI-TOF mass spectrum of TPBT.

**Figure S13.** MALDI-TOF mass spectrum of F-TPBT.


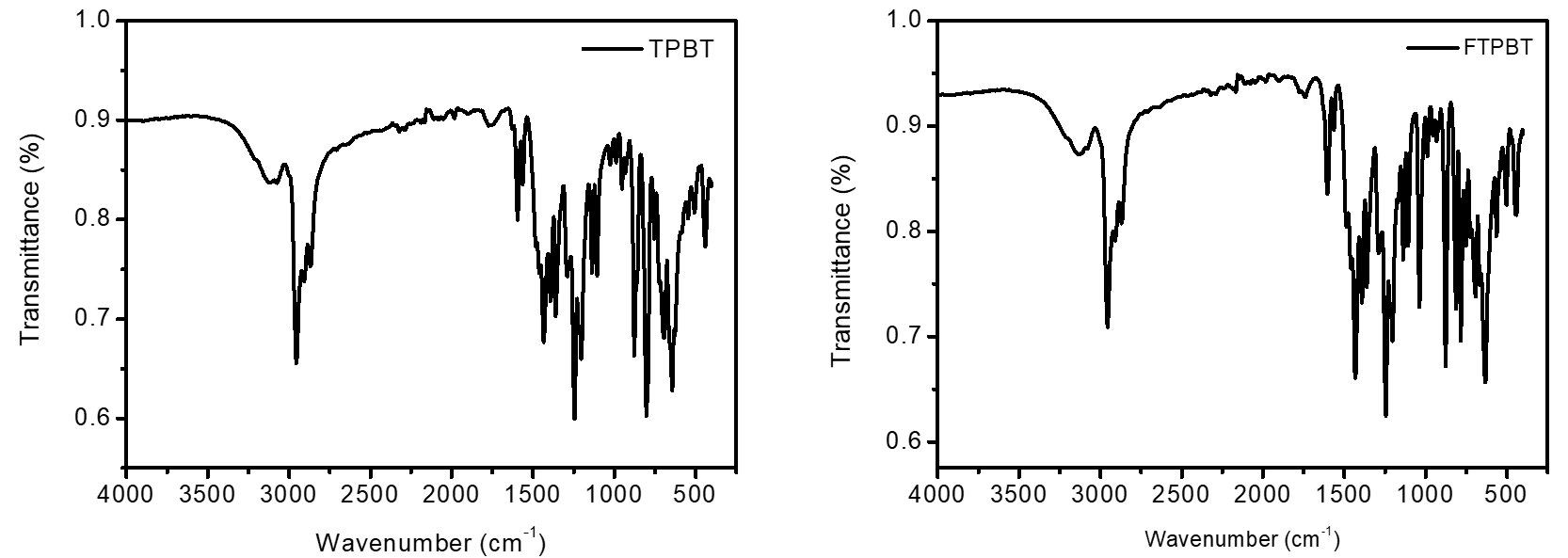


**Figure S14.** FT-IR spectrum of TPBT.


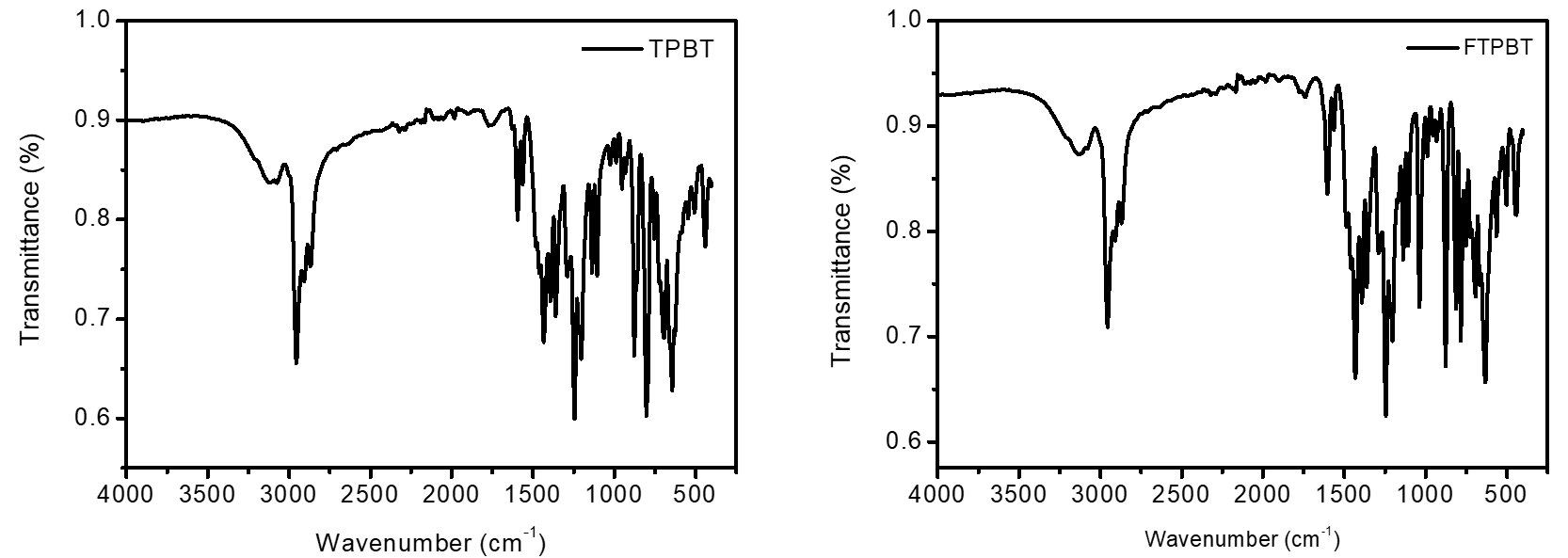


**Figure S15.** FT-IR spectrum of F-TPBT.


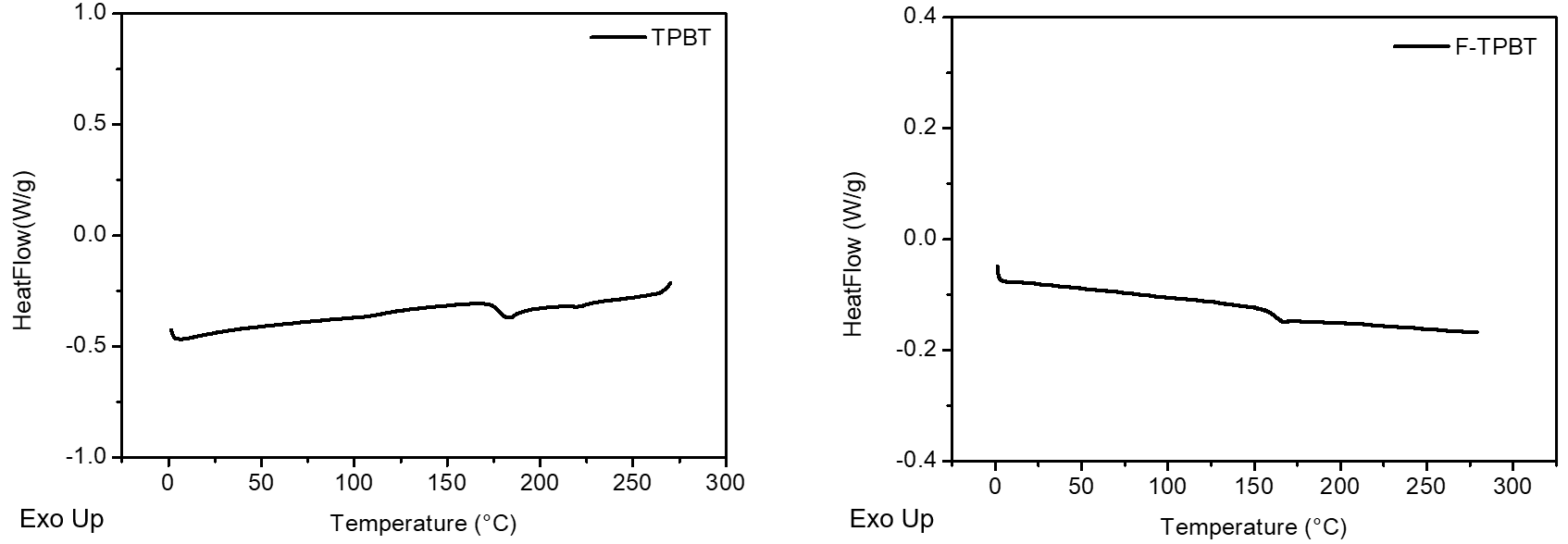


**Figure S16.** DSC curve of TPBT (2^nd^ cycle).


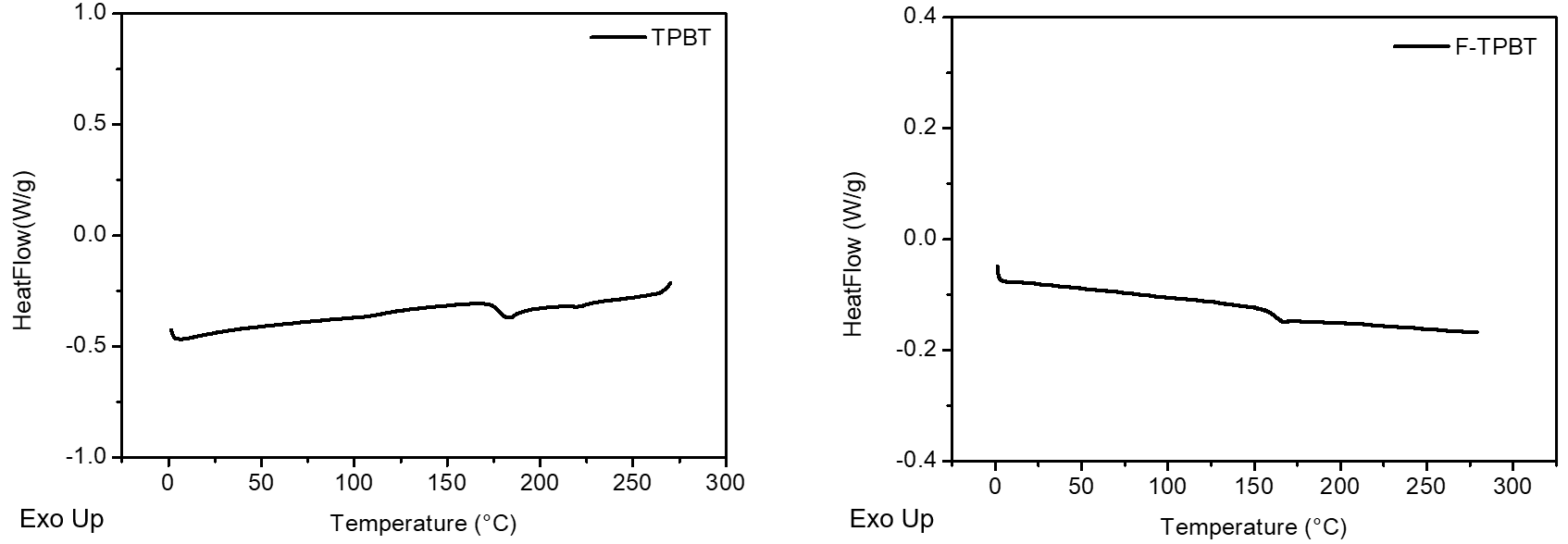


**Figure S17.** DSC curve of F-TPBT (2^nd^ cycle).


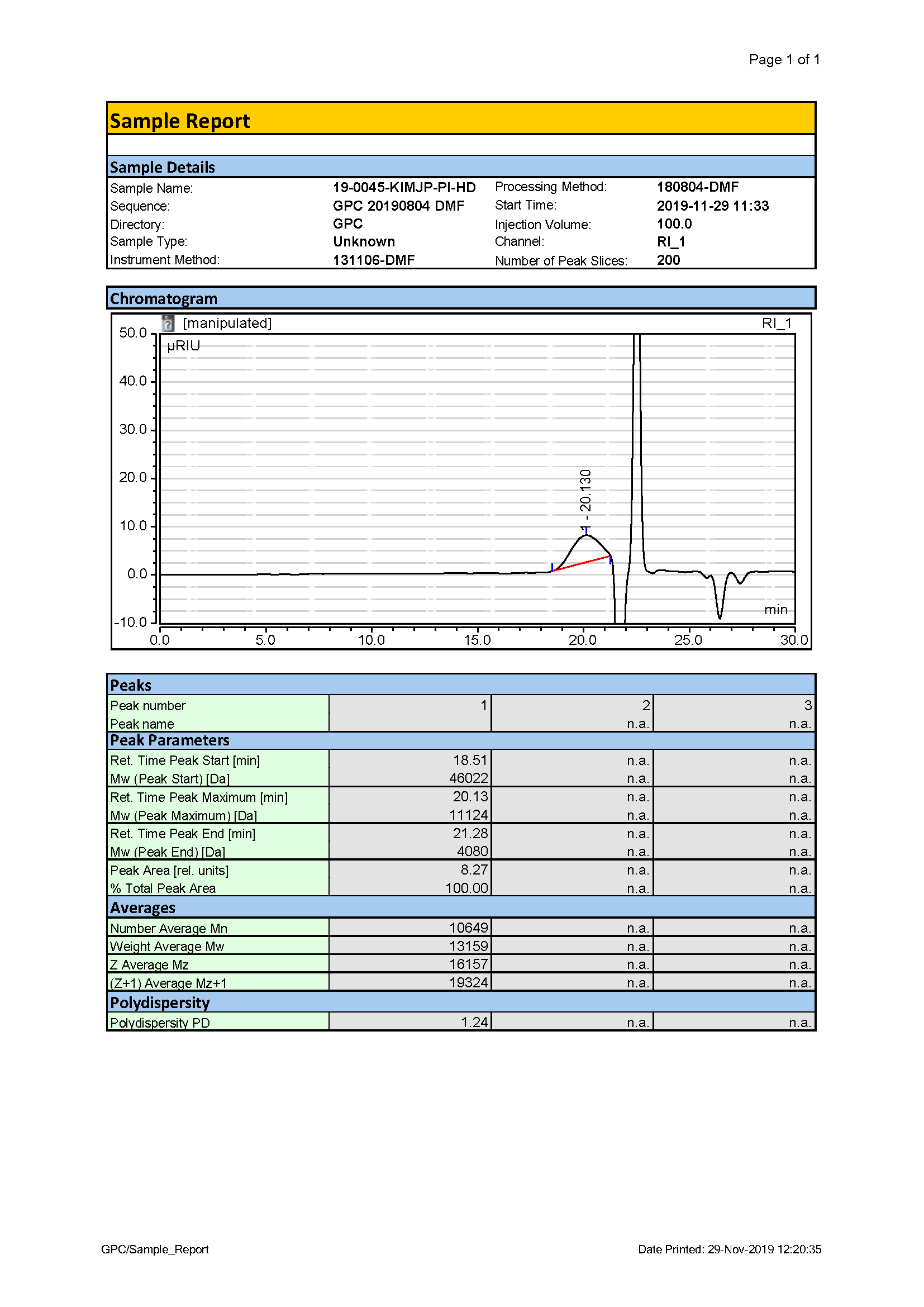


**Figure S18.** Gel permeation chromatography (GPC) trace of PI-HD.

**Table S1.** Parameters extracted from the GPC trace of PI-HD.

| **Peaks** | | | |
| --- | --- | --- | --- |
| Peak number | 1 | 2 | 3 |
| Peak name |  | n.a. | n.a. |
| **Peak Parameters** | | | |
| Ret. time peak start [min]  *M*_w_ (peak start) [Da] | 18.51  46022 | n.a.  n.a. | n.a.  n.a. |
| Ret. time peak maximum [min]  *M*_w_ (peak maximum) [Da] | 20.13  11124 | n.a.  n.a. | n.a.  n.a. |
| Ret. time peak end [min]  *M*_w_ (peak end) [Da] | 21.28  4080 | n.a.  n.a. | n.a.  n.a. |
| Peak area [rel. units]  % Total peak area | 8.27  100.00 | n.a.  n.a. | n.a.  n.a. |
| **Averages** | | | |
| *M*_n_ | 10649 | n.a. | n.a. |
| *M*_w_ | 13159 | n.a. | n.a. |
| Z-average molecular weight | 16157 | n.a. | n.a. |
| (Z+1)-average molecular weight | 19324 | n.a. | n.a. |
| **Polydispersity** | | | |
| Polydispersity | 1.24 | n.a. | n.a. |

1.5 Theoretical calculations

Theoretical calculations and visualizations were carried out using Gaussian 16, GaussView 6.0, and Avogadro. Density functional theory (DFT)-based geometry optimizations of Tinuvin 327 (M), TPBT, and F-TPBT were performed using the WB97XD functional with the def2-tzvp basis set and the IEFPCM (dichloromethane) solvation model. Subsequently, time-dependent DFT (TD-DFT) calculations were conducted on the WB97XD/def2-tzvp optimized structures using the PBE0(=PBE1PBE) functional with the def2-tzvp basis set and the IEFPCM (dichloromethane) solvation model. The potential energy surface (PES) of M in the excited state was explored by varying the O–H distance using TD-DFT-based geometry optimizations (PBE1PBE/def2-tzvp, IEFPCM (dichloromethane) for excited states). The PES of M in the ground state was also explored using the same method.

**1. Optimized geometry result of Tinuvin 327 (M)**

| C | 0 | 2.296592874 | 1.446484 | -0.10605 |
| --- | --- | --- | --- | --- |
| C | 0 | 2.09857477 | 0.74439 | -1.27311 |
| C | 0 | 1.628960247 | -0.56628 | -1.23669 |
| C | 0 | 1.342232489 | -1.21628 | -0.0268 |
| C | 0 | 1.540056413 | -0.50947 | 1.178912 |
| C | 0 | 2.007921685 | 0.789982 | 1.095189 |
| N | 0 | 1.447439956 | -1.23151 | -2.47441 |
| N | 0 | 1.70752306 | -0.64859 | -3.62199 |
| C | 0 | 1.41188943 | -1.59191 | -4.53221 |
| C | 0 | 0.968389125 | -2.74761 | -3.85022 |
| N | 0 | 1.005719463 | -2.47649 | -2.53641 |
| C | 0 | 1.489444558 | -1.55222 | -5.93276 |
| C | 0 | 1.110748018 | -2.69535 | -6.58362 |
| C | 0 | 0.664276528 | -3.8606 | -5.91137 |
| C | 0 | 0.586737057 | -3.90464 | -4.54534 |
| O | 0 | 0.890345752 | -2.47661 | 0.017649 |
| C | 0 | 2.8075548 | 2.880589 | -0.08252 |
| C | 0 | 1.246075805 | -1.16146 | 2.529967 |
| C | 0 | 3.063098263 | 3.418031 | -1.48617 |
| C | 0 | 1.76868908 | 3.777729 | 0.597825 |
| C | 0 | 4.120629282 | 2.938108 | 0.704547 |
| C | 0 | -0.234758663 | -1.55192 | 2.61551 |
| C | 0 | 2.133222736 | -2.39766 | 2.723069 |
| C | 0 | 1.532813595 | -0.21096 | 3.690453 |
| Cl | 0 | 1.171337224 | -2.74473 | -8.31201 |
| H | 0 | 2.297391556 | 1.178797 | -2.24086 |
| H | 0 | 2.160435603 | 1.333554 | 2.016957 |
| H | 0 | 1.827367011 | -0.67317 | -6.46436 |
| H | 0 | 0.383498113 | -4.72201 | -6.50325 |
| H | 0 | 0.246452544 | -4.79209 | -4.02774 |
| H | 0 | 0.815650352 | -2.80348 | -0.91268 |
| H | 0 | 3.427241225 | 4.445841 | -1.42164 |
| H | 0 | 3.818761483 | 2.829901 | -2.0129 |
| H | 0 | 2.151198467 | 3.425455 | -2.08846 |
| H | 0 | 2.12658965 | 4.810333 | 0.630089 |
| H | 0 | 1.570649409 | 3.459825 | 1.623504 |
| H | 0 | 0.823233321 | 3.760056 | 0.05015 |
| H | 0 | 4.882133338 | 2.31108 | 0.234339 |
| H | 0 | 4.494939779 | 3.964851 | 0.737548 |
| H | 0 | 3.989928128 | 2.596188 | 1.733286 |
| H | 0 | -0.439181946 | -2.00201 | 3.59087 |
| H | 0 | -0.868870884 | -0.66739 | 2.51302 |
| H | 0 | -0.511095989 | -2.26658 | 1.842671 |
| H | 0 | 1.927232854 | -2.84766 | 3.698145 |
| H | 0 | 3.189379999 | -2.11679 | 2.697971 |
| H | 0 | 1.956745506 | -3.14767 | 1.95448 |
| H | 0 | 0.912752137 | 0.687929 | 3.647681 |
| H | 0 | 1.307257404 | -0.72359 | 4.628144 |
| H | 0 | 2.582355324 | 0.091826 | 3.723434 |

**2. Optimized geometry result of TPBT**

| C | 0 | 1.344274 | 18.81143 | 25.18801 |
| --- | --- | --- | --- | --- |
| C | 0 | 2.603991 | 18.4637 | 24.75427 |
| C | 0 | 2.767253 | 17.49717 | 23.76466 |
| C | 0 | 1.670718 | 16.84681 | 23.17831 |
| C | 0 | 0.37258 | 17.1927 | 23.61194 |
| C | 0 | 0.256847 | 18.15865 | 24.59673 |
| N | 0 | 4.089051 | 17.18531 | 23.36311 |
| N | 0 | 5.1357 | 17.77863 | 23.88652 |
| C | 0 | 6.175582 | 17.21325 | 23.25148 |
| C | 0 | 5.676337 | 16.26374 | 22.33174 |
| N | 0 | 4.337253 | 16.2745 | 22.4352 |
| C | 0 | 7.557114 | 17.42825 | 23.37929 |
| C | 0 | 8.376608 | 16.67672 | 22.58232 |
| C | 0 | 7.882853 | 15.71442 | 21.65124 |
| C | 0 | 6.527201 | 15.50264 | 21.52094 |
| O | 0 | 1.818271 | 15.91551 | 22.22703 |
| C | 0 | 1.102731 | 19.85961 | 26.26664 |
| C | 0 | -0.86267 | 16.52049 | 23.01079 |
| C | 0 | 2.406341 | 20.45091 | 26.7903 |
| C | 0 | 0.252591 | 20.99499 | 25.68895 |
| C | 0 | 0.355838 | 19.21765 | 27.43952 |
| C | 0 | -0.93528 | 16.79898 | 21.50469 |
| C | 0 | -0.82103 | 15.00932 | 23.26757 |
| C | 0 | -2.1527 | 17.04976 | 23.63275 |
| C | 0 | 17.61827 | 7.694289 | 21.64976 |
| C | 0 | 16.64938 | 8.667544 | 21.54873 |
| C | 0 | 16.78324 | 9.870273 | 22.23842 |
| C | 0 | 17.89426 | 10.13541 | 23.05355 |
| C | 0 | 18.89681 | 9.148154 | 23.16649 |
| C | 0 | 18.72311 | 7.968227 | 22.46366 |
| N | 0 | 15.75255 | 10.83024 | 22.09014 |
| N | 0 | 14.71416 | 10.62518 | 21.31492 |
| C | 0 | 13.9876 | 11.74927 | 21.44708 |
| C | 0 | 14.66087 | 12.61098 | 22.34185 |
| N | 0 | 15.78445 | 11.98885 | 22.7295 |
| C | 0 | 12.76691 | 12.11246 | 20.86274 |
| C | 0 | 12.24348 | 13.3439 | 21.19159 |
| C | 0 | 12.93629 | 14.20214 | 22.09715 |
| C | 0 | 14.12984 | 13.86584 | 22.67587 |
| O | 0 | 18.03086 | 11.28847 | 23.72145 |
| C | 0 | 17.52465 | 6.358628 | 20.92312 |
| C | 0 | 20.13272 | 9.378178 | 24.03759 |
| C | 0 | 16.25862 | 6.255121 | 20.08066 |
| C | 0 | 18.73525 | 6.195075 | 19.99936 |
| C | 0 | 17.51803 | 5.222817 | 21.95052 |
| C | 0 | 20.9203 | 10.59118 | 23.52801 |
| C | 0 | 19.71805 | 9.592673 | 25.49815 |
| C | 0 | 21.07749 | 8.17919 | 24.00608 |
| C | 0 | 5.71277 | 15.82675 | 10.6764 |
| C | 0 | 6.301454 | 15.45608 | 11.86475 |
| C | 0 | 6.930122 | 14.21893 | 11.98461 |
| C | 0 | 6.989589 | 13.31296 | 10.91462 |
| C | 0 | 6.390441 | 13.67824 | 9.689789 |
| C | 0 | 5.776177 | 14.91658 | 9.615417 |
| N | 0 | 7.513191 | 13.89744 | 13.23462 |
| N | 0 | 7.473068 | 14.72036 | 14.25556 |
| C | 0 | 8.118835 | 14.05519 | 15.22997 |
| C | 0 | 8.535982 | 12.80733 | 14.71499 |
| N | 0 | 8.129404 | 12.74372 | 13.43814 |
| C | 0 | 8.397343 | 14.43044 | 16.55102 |
| C | 0 | 9.093155 | 13.53596 | 17.33489 |
| C | 0 | 9.506231 | 12.27913 | 16.79946 |
| C | 0 | 9.243076 | 11.89524 | 15.51267 |
| O | 0 | 7.592686 | 12.12156 | 11.02053 |
| C | 0 | 5.009591 | 17.16469 | 10.48615 |
| C | 0 | 6.419192 | 12.73379 | 8.487103 |
| C | 0 | 5.050742 | 18.01591 | 11.74985 |
| C | 0 | 5.69225 | 17.94117 | 9.356282 |
| C | 0 | 3.544402 | 16.91852 | 10.11435 |
| C | 0 | 7.867565 | 12.4495 | 8.071696 |
| C | 0 | 5.70012 | 11.42289 | 8.827352 |
| C | 0 | 5.711098 | 13.33831 | 7.27701 |
| C | 0 | 8.534027 | 14.60689 | 19.51551 |
| C | 0 | 9.419398 | 13.86485 | 18.73743 |
| C | 0 | 10.62829 | 13.45945 | 19.29787 |
| C | 0 | 10.96119 | 13.78902 | 20.60973 |
| C | 0 | 10.06166 | 14.53886 | 21.3636 |
| C | 0 | 8.841856 | 14.94888 | 20.82959 |
| H | 0 | 3.49207 | 18.92244 | 25.16235 |
| H | 0 | -0.73512 | 18.4271 | 24.93222 |
| H | 0 | 7.951761 | 18.16117 | 24.07229 |
| H | 0 | 9.44808 | 16.82811 | 22.64022 |
| H | 0 | 6.13029 | 14.76196 | 20.83779 |
| H | 0 | 2.788129 | 15.80682 | 22.05903 |
| H | 0 | 2.184915 | 21.19459 | 27.55995 |
| H | 0 | 3.046395 | 19.68719 | 27.24022 |
| H | 0 | 2.972529 | 20.94984 | 25.99917 |
| H | 0 | 0.065252 | 21.75255 | 26.45551 |
| H | 0 | -0.71478 | 20.63363 | 25.33226 |
| H | 0 | 0.763316 | 21.47721 | 24.85098 |
| H | 0 | -0.60804 | 18.80791 | 27.12867 |
| H | 0 | 0.942546 | 18.40591 | 27.8782 |
| H | 0 | 0.167839 | 19.96207 | 28.21868 |
| H | 0 | -1.83216 | 16.33139 | 21.08801 |
| H | 0 | -0.99856 | 17.87438 | 21.31577 |
| H | 0 | -0.06849 | 16.40738 | 20.97435 |
| H | 0 | -1.71948 | 14.54258 | 22.85333 |
| H | 0 | -0.79997 | 14.80294 | 24.34142 |
| H | 0 | 0.049028 | 14.54048 | 22.8104 |
| H | 0 | -2.19902 | 16.85946 | 24.70826 |
| H | 0 | -2.28081 | 18.12244 | 23.46551 |
| H | 0 | -3.00069 | 16.53923 | 23.16974 |
| H | 0 | 15.76737 | 8.530397 | 20.94135 |
| H | 0 | 19.4873 | 7.208375 | 22.54852 |
| H | 0 | 12.24954 | 11.43483 | 20.19486 |
| H | 0 | 12.50338 | 15.17136 | 22.31493 |
| H | 0 | 14.64466 | 14.53741 | 23.35171 |
| H | 0 | 17.2342 | 11.84421 | 23.5301 |
| H | 0 | 16.23467 | 5.284831 | 19.57829 |
| H | 0 | 15.35601 | 6.334873 | 20.69237 |
| H | 0 | 16.21972 | 7.029811 | 19.31009 |
| H | 0 | 18.6804 | 5.236857 | 19.47461 |
| H | 0 | 19.67506 | 6.21763 | 20.55592 |
| H | 0 | 18.76573 | 6.991906 | 19.25124 |
| H | 0 | 18.4253 | 5.224442 | 22.55918 |
| H | 0 | 16.65972 | 5.309469 | 22.62235 |
| H | 0 | 17.45702 | 4.25586 | 21.44265 |
| H | 0 | 21.81308 | 10.73352 | 24.14393 |
| H | 0 | 21.24605 | 10.43142 | 22.49625 |
| H | 0 | 20.33102 | 11.50623 | 23.56454 |
| H | 0 | 20.61001 | 9.735374 | 26.11516 |
| H | 0 | 19.1833 | 8.717448 | 25.87784 |
| H | 0 | 19.07742 | 10.46521 | 25.61715 |
| H | 0 | 20.60399 | 7.271331 | 24.389 |
| H | 0 | 21.45199 | 7.976334 | 22.99923 |
| H | 0 | 21.93964 | 8.395224 | 24.64184 |
| H | 0 | 6.293429 | 16.10339 | 12.72878 |
| H | 0 | 5.315612 | 15.20022 | 8.67942 |
| H | 0 | 8.092457 | 15.39962 | 16.92615 |
| H | 0 | 10.0329 | 11.59345 | 17.45265 |
| H | 0 | 9.556783 | 10.93192 | 15.12995 |
| H | 0 | 7.947435 | 12.04395 | 11.94158 |
| H | 0 | 4.5373 | 18.96319 | 11.56681 |
| H | 0 | 4.550278 | 17.52324 | 12.58777 |
| H | 0 | 6.076267 | 18.24621 | 12.05082 |
| H | 0 | 5.192841 | 18.90262 | 9.205356 |
| H | 0 | 5.65656 | 17.39407 | 8.411325 |
| H | 0 | 6.741327 | 18.13598 | 9.595121 |
| H | 0 | 3.454257 | 16.34241 | 9.190504 |
| H | 0 | 3.027592 | 16.37019 | 10.90668 |
| H | 0 | 3.028079 | 17.87152 | 9.966628 |
| H | 0 | 7.875762 | 11.79086 | 7.198313 |
| H | 0 | 8.377576 | 13.37732 | 7.797504 |
| H | 0 | 8.433981 | 11.9682 | 8.867541 |
| H | 0 | 5.706315 | 10.76402 | 7.95413 |
| H | 0 | 4.657417 | 11.61529 | 9.095516 |
| H | 0 | 6.176451 | 10.89825 | 9.654263 |
| H | 0 | 4.655742 | 13.54196 | 7.476709 |
| H | 0 | 6.185335 | 14.26487 | 6.942873 |
| H | 0 | 5.759392 | 12.62561 | 6.450051 |
| H | 0 | 7.590282 | 14.92646 | 19.08938 |
| H | 0 | 11.34324 | 12.91655 | 18.69101 |
| H | 0 | 10.29278 | 14.76577 | 22.39756 |

**3. Optimized geometry result of F-TPBT**

| C | 0 | -3.6462 | 9.602421 | 0.755854 |
| --- | --- | --- | --- | --- |
| C | 0 | -2.92567 | 8.436558 | 0.88192 |
| C | 0 | -3.11291 | 7.390714 | -0.0186 |
| C | 0 | -4.02979 | 7.480841 | -1.07686 |
| C | 0 | -4.77656 | 8.670197 | -1.21951 |
| C | 0 | -4.55833 | 9.68249 | -0.30186 |
| N | 0 | -2.33985 | 6.218232 | 0.16853 |
| N | 0 | -1.47971 | 6.100985 | 1.15284 |
| C | 0 | -0.96508 | 4.871389 | 0.98231 |
| C | 0 | -1.57347 | 4.285475 | -0.15023 |
| N | 0 | -2.44666 | 5.17884 | -0.64356 |
| C | 0 | 0.003407 | 4.172297 | 1.719725 |
| C | 0 | 0.323178 | 2.914847 | 1.28637 |
| C | 0 | -0.28612 | 2.320155 | 0.143694 |
| C | 0 | -1.23803 | 2.996878 | -0.58347 |
| O | 0 | -4.21586 | 6.480368 | -1.94794 |
| C | 0 | -3.48644 | 10.77897 | 1.709026 |
| C | 0 | -5.79127 | 8.830466 | -2.3514 |
| C | 0 | -2.44376 | 10.50256 | 2.786424 |
| C | 0 | -3.04987 | 12.01685 | 0.91903 |
| C | 0 | -4.82752 | 11.06183 | 2.393599 |
| C | 0 | -5.08824 | 8.716526 | -3.70986 |
| C | 0 | -6.88811 | 7.765105 | -2.23072 |
| C | 0 | -6.47633 | 10.19467 | -2.30784 |
| C | 0 | -7.52816 | -6.31125 | 2.072539 |
| C | 0 | -6.46136 | -5.60576 | 1.564008 |
| C | 0 | -5.98247 | -5.87615 | 0.284453 |
| C | 0 | -6.5589 | -6.8644 | -0.52779 |
| C | 0 | -7.65409 | -7.59645 | -0.02059 |
| C | 0 | -8.09636 | -7.29411 | 1.255118 |
| N | 0 | -4.88221 | -5.11272 | -0.17843 |
| N | 0 | -4.32115 | -4.18312 | 0.556926 |
| C | 0 | -3.33881 | -3.70494 | -0.22772 |
| C | 0 | -3.36353 | -4.40901 | -1.45234 |
| N | 0 | -4.36428 | -5.30021 | -1.38277 |
| C | 0 | -2.39637 | -2.69648 | 0.014631 |
| C | 0 | -1.49732 | -2.42047 | -0.98898 |
| C | 0 | -1.53256 | -3.13594 | -2.22076 |
| C | 0 | -2.44595 | -4.12264 | -2.47387 |
| O | 0 | -6.10484 | -7.13148 | -1.7594 |
| C | 0 | -8.0958 | -6.06106 | 3.463061 |
| C | 0 | -8.32612 | -8.68625 | -0.85577 |
| C | 0 | -7.34359 | -4.95562 | 4.195613 |
| C | 0 | -9.56659 | -5.64925 | 3.343911 |
| C | 0 | -7.995 | -7.34621 | 4.290909 |
| C | 0 | -8.89931 | -8.08502 | -2.14519 |
| C | 0 | -7.31716 | -9.79242 | -1.18885 |
| C | 0 | -9.48488 | -9.33961 | -0.10557 |
| C | 0 | 10.57049 | -0.79265 | 1.763 |
| C | 0 | 9.276187 | -0.80825 | 1.295218 |
| C | 0 | 8.987851 | -1.32576 | 0.03475 |
| C | 0 | 9.989955 | -1.84467 | -0.79903 |
| C | 0 | 11.32252 | -1.83598 | -0.3334 |
| C | 0 | 11.56243 | -1.31286 | 0.924865 |
| N | 0 | 7.634748 | -1.31177 | -0.38539 |
| N | 0 | 6.675013 | -0.83987 | 0.373557 |
| C | 0 | 5.568282 | -1.0023 | -0.37345 |
| C | 0 | 5.941472 | -1.59348 | -1.60113 |
| N | 0 | 7.270574 | -1.77565 | -1.57116 |
| C | 0 | 4.232388 | -0.68554 | -0.09319 |
| C | 0 | 3.299779 | -0.97295 | -1.0626 |
| C | 0 | 3.687926 | -1.5677 | -2.29801 |
| C | 0 | 4.987378 | -1.88301 | -2.58776 |
| O | 0 | 9.721926 | -2.34385 | -2.01282 |
| C | 0 | 10.94738 | -0.23925 | 3.130514 |
| C | 0 | 12.45994 | -2.38656 | -1.19363 |
| C | 0 | 9.731233 | 0.277535 | 3.891072 |
| C | 0 | 11.60458 | -1.34532 | 3.962171 |
| C | 0 | 11.93614 | 0.917633 | 2.954397 |
| C | 0 | 12.22446 | -3.87279 | -1.49075 |
| C | 0 | 12.56264 | -1.59359 | -2.5026 |
| C | 0 | 13.80918 | -2.27281 | -0.48723 |
| C | 0 | 1.443512 | 0.608515 | -0.45991 |
| C | 0 | 1.874225 | -0.65704 | -0.83896 |
| C | 0 | 0.873983 | -1.6078 | -1.0005 |
| C | 0 | -0.4801 | -1.36396 | -0.80914 |
| C | 0 | -0.81186 | -0.06744 | -0.43346 |
| C | 0 | 0.113826 | 0.952553 | -0.24736 |
| H | 0 | -2.20241 | 8.295428 | 1.670483 |
| H | 0 | -5.12876 | 10.59439 | -0.40818 |
| H | 0 | 0.471109 | 4.60893 | 2.592797 |
| H | 0 | 1.059859 | 2.337778 | 1.831301 |
| H | 0 | -1.70536 | 2.559011 | -1.45576 |
| H | 0 | -3.61012 | 5.741055 | -1.69141 |
| H | 0 | -2.3614 | 11.37055 | 3.444429 |
| H | 0 | -2.71754 | 9.642638 | 3.40279 |
| H | 0 | -1.45683 | 10.31675 | 2.35535 |
| H | 0 | -2.94019 | 12.87243 | 1.59089 |
| H | 0 | -3.77988 | 12.28488 | 0.152474 |
| H | 0 | -2.0902 | 11.84348 | 0.425943 |
| H | 0 | -5.60796 | 11.30103 | 1.668265 |
| H | 0 | -5.15782 | 10.1955 | 2.971983 |
| H | 0 | -4.73063 | 11.9117 | 3.074608 |
| H | 0 | -5.81898 | 8.848246 | -4.51258 |
| H | 0 | -4.32964 | 9.496807 | -3.81329 |
| H | 0 | -4.60794 | 7.748364 | -3.83775 |
| H | 0 | -7.61776 | 7.897948 | -3.03425 |
| H | 0 | -7.41423 | 7.865618 | -1.27778 |
| H | 0 | -6.48317 | 6.757142 | -2.29739 |
| H | 0 | -7.03346 | 10.34497 | -1.37977 |
| H | 0 | -5.76411 | 11.01558 | -2.42261 |
| H | 0 | -7.18796 | 10.2567 | -3.13411 |
| H | 0 | -5.96789 | -4.83082 | 2.130427 |
| H | 0 | -8.93512 | -7.8525 | 1.646117 |
| H | 0 | -2.37863 | -2.16457 | 0.956921 |
| H | 0 | -0.80992 | -2.8754 | -2.9841 |
| H | 0 | -2.46313 | -4.65346 | -3.41693 |
| H | 0 | -5.3447 | -6.52362 | -1.93841 |
| H | 0 | -7.78264 | -4.8107 | 5.185273 |
| H | 0 | -6.28891 | -5.20685 | 4.333062 |
| H | 0 | -7.40383 | -4.00365 | 3.662254 |
| H | 0 | -9.99002 | -5.47459 | 4.336696 |
| H | 0 | -10.1639 | -6.42256 | 2.856533 |
| H | 0 | -9.66573 | -4.72915 | 2.76276 |
| H | 0 | -8.548 | -8.16746 | 3.83029 |
| H | 0 | -6.95312 | -7.65814 | 4.397339 |
| H | 0 | -8.40755 | -7.18331 | 5.290263 |
| H | 0 | -9.39252 | -8.8687 | -2.7268 |
| H | 0 | -9.6442 | -7.32 | -1.91088 |
| H | 0 | -8.12422 | -7.63514 | -2.76263 |
| H | 0 | -7.8107 | -10.5749 | -1.77189 |
| H | 0 | -6.9331 | -10.247 | -0.27189 |
| H | 0 | -6.47556 | -9.41337 | -1.76544 |
| H | 0 | -9.1551 | -9.82635 | 0.815626 |
| H | 0 | -10.2703 | -8.62137 | 0.142557 |
| H | 0 | -9.9283 | -10.1068 | -0.74419 |
| H | 0 | 8.45363 | -0.42563 | 1.879915 |
| H | 0 | 12.58174 | -1.3047 | 1.284149 |
| H | 0 | 3.951668 | -0.23909 | 0.851832 |
| H | 0 | 2.918977 | -1.76584 | -3.0343 |
| H | 0 | 5.266811 | -2.32976 | -3.53316 |
| H | 0 | 8.747353 | -2.26189 | -2.16368 |
| H | 0 | 10.04669 | 0.663318 | 4.86309 |
| H | 0 | 9.235073 | 1.090747 | 3.355456 |
| H | 0 | 8.999327 | -0.51434 | 4.068949 |
| H | 0 | 11.88811 | -0.95894 | 4.944899 |
| H | 0 | 12.50611 | -1.73034 | 3.481145 |
| H | 0 | 10.91615 | -2.18127 | 4.108511 |
| H | 0 | 12.84668 | 0.596085 | 2.444603 |
| H | 0 | 11.48829 | 1.724677 | 2.369358 |
| H | 0 | 12.22239 | 1.319842 | 3.929955 |
| H | 0 | 13.05088 | -4.26153 | -2.09211 |
| H | 0 | 12.18558 | -4.4461 | -0.5608 |
| H | 0 | 11.29664 | -4.0352 | -2.03593 |
| H | 0 | 13.39024 | -1.98246 | -3.10223 |
| H | 0 | 12.76321 | -0.53922 | -2.29502 |
| H | 0 | 11.6499 | -1.66242 | -3.09128 |
| H | 0 | 14.07198 | -1.23475 | -0.26889 |
| H | 0 | 13.83351 | -2.84131 | 0.445788 |
| H | 0 | 14.58343 | -2.67893 | -1.14187 |
| F | 0 | -2.10074 | 0.215352 | -0.23849 |
| F | 0 | 1.242725 | -2.85033 | -1.32166 |
| F | 0 | 2.360328 | 1.56966 | -0.33052 |

**Reference**

1. Yu, H.C., J.W. Jung, J.Y. Choi, and C.M. Chung, *Kinetic study of low‐temperature imidization of poly(amic acid)s and preparation of colorless, transparent polyimide films.* J. Polym. Sci. Part A: Polym. Chem., 2015, **54**(11), 1593-1602.
